# Role of the Hippo pathway in liver regeneration and repair: recent advances

**DOI:** 10.1186/s41232-022-00235-5

**Published:** 2022-12-05

**Authors:** Monica Pibiri, Gabriella Simbula

**Affiliations:** Department of Biomedical Sciences, Oncology and Molecular Pathology Unit, University of Cagliari, Cittadella Universitaria di Monserrato, S.P. Monserrato-Sestu km 0.700, Blocco A. 09042 Monserrato, Cagliari, Italy

**Keywords:** Hippo pathway, 2/3 PH, YAP, TAZ, IRI, NAFLD, NASH, Liver fibrosis, Hepatocarcinogenesis

## Abstract

Although the signaling pathways involved in normal liver regeneration have been well characterized, less has been done for livers affected by chronic tissue damage. These “abnormal livers” have an impaired regenerative response that leads to liver repair and fibrosis. The tumor suppressor Hippo pathway plays a key role in liver regeneration and repair. On this basis, this review discusses recent studies focusing on the involvement of the Hippo signaling pathway during “normal healthy liver regeneration” (i.e., in a normal liver after 2/3 partial hepatectomy) and “abnormal liver regeneration” (i.e., in a liver damaged by chronic disease). This could be an important question to address with respect to new therapies aimed at improving impaired liver regenerative responses. The studies reported here have shown that activation of the Hippo coactivators YAP/TAZ during normal liver regeneration promotes the formation of a new bile duct network through direct BEC proliferation or/and hepatocyte dedifferentiation to HPCs which can trans-differentiate to BECs. Moreover, YAP/TAZ signaling interaction with other signaling pathways mediates the recruitment and activation of Kupffer cells, which release mitogenic cytokines for parenchymal and/or non-parenchymal cells and engage in phagocytosis of cellular debris. In addition, YAP-mediated activation of stellate cells (HSCs) promotes liver regeneration through the synthesis of extracellular matrix. However, in chronically diseased livers, where the predetermined threshold for proper liver regeneration is exceeded, YAP/TAZ activation results in a reparative process characterized by liver fibrosis. In this condition, YAP/TAZ activation in parenchymal and non-parenchymal cells results in (i) differentiation of quiescent HSCs into myofibroblastic HSCs; (ii) recruitment of macrophages releasing inflammatory cytokines; (iii) polarization of macrophages toward the M2 phenotype. Since accumulation of damaged hepatocytes in chronic liver injury represent a significant risk factor for the development of hepatocarcinoma, this review also discussed the involvement of the Hippo pathway in the clearance of damaged cells.

## Introduction

### Liver regeneration and repair

Characterization of the molecular mechanisms associated with regeneration and repair after tissue injury is central to the development of therapeutic strategies aimed at improving the outcomes of acute and chronic liver injury.

Liver regeneration has been studied for many decades, and the mechanisms underlying the renewal of a normal liver after resection or moderate tissue injury are well described. Adult hepatocytes are characterized by a very low replication rate but can rapidly re-enter the cell cycle after tissue loss or death [1–4]. The pioneering study of Bucher and Swaffield [5] showed that the extent of hepatocyte proliferation in the regenerating liver of adult rats depends on the size of the resected tissue, for resections involving 40–70% of the liver. When 30% of the liver mass is resected, a significant wave of DNA replication is no longer possible. In agreement with this, Miyaoka et al. [6] reported that after 1/3 partial hepatectomy (1/3 PH), normal liver volume is essentially restored by hypertrophy. However, after 2/3 PH, hypertrophy followed by cell proliferation occurs, and the two processes lead to the restoration of liver size with hyperplasia representing the major contributor to liver mass recovery [6, 7]. Therefore, the surgical resection of a maximum of 70% of the organ mass (2/3 PH) represents the best experimental model to study liver regeneration in rodents [8].

**Fig. 1:**
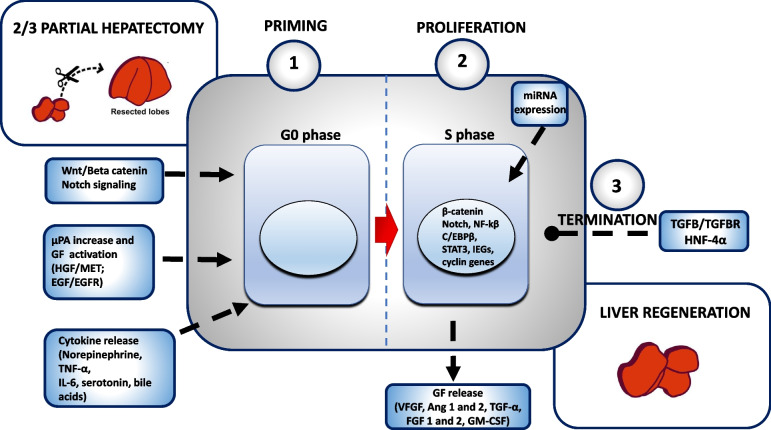
Schematic overview of liver regeneration after 2/3 PH. After partial hepatectomy, the remaining liver cells proliferate until the original organ size is restored. Three phases of liver regeneration have been identified: (1) priming, (2) proliferation, and (3) termination. The priming phase (1) is related to the activation of growth factors (HGF and EGF, which are ligands of MET and EGF receptors, respectively), induced by the 2/3 PH-induced increase in μPA and the nuclear translocation of Notch1 and beta-catenin into hepatocytes, as well as by the release of cytokines (TNF-α; norepinephrine, bile acids, IL-6, serotonin) that modulate hepatocyte proliferation and the interaction between hepatocytes and non-parenchymal cells. The proliferation phase (2) is preceded by activation and nuclear translocation of transcription factors such as STAT3, C/EBPβ, and NFκB. Increased expression of IEGs (c-Fos, c-Jun, and c-Myc) is also observed. All these factors promote the proliferation phase, leading to the transcription of cyclin genes. Proliferating hepatocytes release many growth factors that stimulate the proliferation of non-parenchymal cells: VEGF and Ang1 and 2, mitogenic for LSECs; TGF-α, mitogenic for endothelial cells, LSECs, and HSCs; FGF1 and 2, mitogenic for HSCs and LSECs; and GM-CSF, which stimulates the proliferation of KCs. In addition, microRNAs (miRNAs) were also found to be involved in the regulation of hepatocyte DNA synthesis in mouse models of liver regeneration. The termination phase (3) is likely promoted by activation of signal transduction pathways that suppress cell growth, such as that mediated by TGF-β/TGFβ receptor and HNF-4α

In response to 2/3 PH, the remaining cells proliferate until the original organ size is restored (within 7 to 10 days). Although the exact molecular events associated with the G0/G1 transition are not fully elucidated, three phases that control liver regeneration have been identified: (i) priming, which is associated with growth factor activation and cytokine release, (ii) proliferation, which is promoted by immediate early gene/transcription factor activation, and (iii) termination, which is likely controlled by signal transduction pathways that lead to inhibition of the regenerative response [2] (Fig. 1). More specifically, the priming phase is promoted by the 2/3 PH-induced increase in urokinase plasminogen activator (μPA) and nuclear translocation of Notch1 and β-catenin in hepatocytes within 15–20 min [2, 9]. Then, there is an increase in active hepatocyte growth factor (HGF) in blood promoted by μPA [10–12], followed by activation of HGF and epidermal growth factor (EGF) receptors (R) within 30–60 min after surgery [13]. Several EGFR ligands have been found to promote liver regeneration, e.g., EGF produced by Brunner glands in the intestine, TGF-α induced by ADAM17-mediated cleavage, amphiregulin regulated by YAP, and heparin-binding EGF-like growth factor (HB-EGF) [14]. The latter, produced by macrophages and endothelial cells, has been identified as the causative factor for hepatocyte hypertrophy observed after less than 30% of liver resections [15]. In addition, combined EGFR and MET signaling has been shown to be a key regulator of normal hepatocyte function and liver regeneration [16, 17]. Shortly after 2/3 PH, there is also a rapid increase in blood concentrations of norepinephrine, tumor necrosis factor (TNF)-α, interleukin (IL) 6, serotonin, and bile acids [1–3, 18–21]. These factors regulate and optimize the timing and intensity of intracellular signals important for hepatocyte proliferation and their interactions with paracrine cells [9]. Proliferating hepatocytes release many growth factors: vascular endothelial growth factor (VEGF) and angiopoietins (Ang) 1 and 2, mitogenic for liver sinusoidal endothelial cells (LSECs); transforming growth factor α (TGF-α), mitogenic for endothelial cells, LSECs, and stellate cells (HSCs); fibroblast growth factor 1 and 2 (FGF1 and FGF2), mitogenic for HSCs and LSECs; and granulocyte-macrophage colony-stimulating factor (GM-CSF), which stimulates the proliferation of Kupffer cells (KCs). Thus, hepatocytes orchestrate liver regeneration to enable the formation of histologically complete liver tissue [14]. Hepatocyte DNA synthesis is preceded by the activation and nuclear translocation of transcription factors such as Signal Transducer and Activator of Transcription 3 (STAT3), CCAAT/enhancer-binding protein beta (C/EBPβ), and Nuclear Factor Kappa B (NFκB) [2]. In addition, increased expression of cell cycle inhibitors (p21 and p53), immediate early genes (IEGs) (c-Fos, c-Jun, and c-Myc), transforming growth factor beta (TGF-β) [1], and transcription factors (Octamer 4 and Nanog) [22] is also observed. These factors promote the proliferation phase, leading to the transcription of delayed early genes encoding cell cycle regulatory proteins, namely cyclins [23–25]. Moreover, microRNAs (miRNAs) have been found to be involved in the regulation of hepatocyte DNA synthesis in mouse models [26]. Accordingly, (i) expression changes in seven miRNAs were identified in mouse liver tissue 36 h after 2/3 PH [26]; (ii) in the early phase of liver regeneration, a negative feedback mechanism was observed between miRNA expression and maturation processing target genes, which may be related to the regulation of the steady-state level of liver regeneration [27]; and (iii) upregulation of miRNA-221 and miRNA-21 and downregulation of miRNA-26a were found to promote liver regeneration [27]. In addition, recent studies have shown that the coordinated expression of miRNAs also plays a role in human liver regeneration, although further analysis is required [28]. Recently, by using the hepatocyte-specific ProTracer system in 2/3 PH mice, it was shown that the periportal hepatocytes were the first to proliferate after surgery, followed by the midlobular and pericentral hepatocytes [29]. By providing a high-resolution and cumulative record of the major proliferation events in hepatocytes during liver regeneration, the ProTacer genetic approach has allowed us to confirm the regeneration wave from portal to central hepatocytes, which has been proposed in previous studies [30–34]. The proliferation response of hepatocytes stops immediately when the original liver mass is restored, i.e., during the termination phase [9, 35]. This event is likely promoted by the activation of signal transduction pathways associated with cell growth inhibition, such as that mediated by transforming growth factor beta (TGF-β)/TGFβ receptor (R) [2]. Furthermore, a recent study showed a key role for hepatocyte nuclear factor 4 α (HNF4α) in termination of liver regeneration and recovery of hepatocyte function [36]. HNF4α plays a key role in maintaining hepatocyte differentiation and function during embryogenesis and homeostasis. Therefore, its re-expression following initial decline represent a crucial event for hepatocytes to exit the cell cycle and recover in the termination phase of liver regeneration after acute liver injury.

**Fig. 2:**
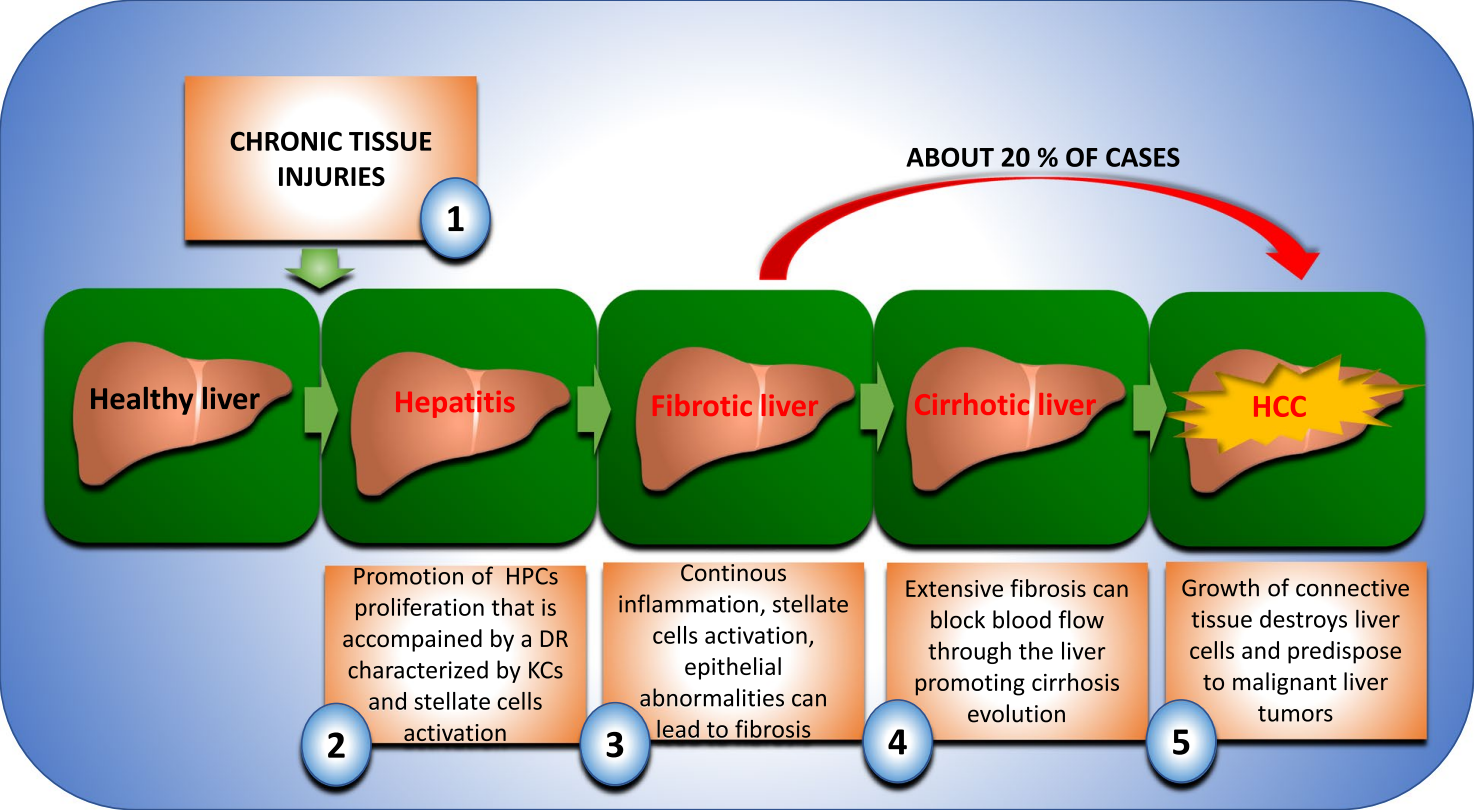
Schematic representation of the events involved in the progression of liver injury. (1) Chronic tissue damage significantly impairs the regenerative capacity of the liver. Therefore, activation of secondary proliferation pathways characterized by proliferation of hepatocyte progenitor cells (HPCs) occurs. These cells are the source of hepatocyte, cholangiocytes, and drainage tubule regeneration. The proliferation of HPCs is accompanied by a ductular reaction (DR) that leads to the recruitment of macrophages (KCs) and stellate cells, resulting in persistent inflammation (2). Persistent inflammation, stellate cell activation, and epithelial abnormalities may lead to fibrosis (3). Extensive liver fibrosis can block the blood flow through the liver, promoting cirrhosis evolution (4). Cirrhosis can be defined as the final stage of fibrosis and is associated with significant changes in liver architecture that predispose to malignant liver tumors (HCC) (5). As shown in the figure (red arrow), approximatively about 20% of cases of HCC may develop in a non-cirrhotic liver, suggesting multiple mechanisms of hepatocarcinogenesis

While many studies have focused on signaling pathways involved in normal liver regeneration, fewer studies have been performed for abnormal livers [37], such as those affected by chronic tissue injury. The latter significantly impairs the regenerative response of the liver through excessive inflammation, scarring, and epithelial abnormalities. Whereas in a normal liver the replacement of necrotic hepatocytes occurs by replication of the cells remaining in the lobules, this primary pathway is impaired in chronically damaged livers. Therefore, activation of secondary proliferation pathways occurs. As a result, proliferation of bipotent cells occurs, namely hepatic progenitor cells (HPCs), which are mainly located in the periportal region of the liver [38–40]. These cells are the source of regenerating hepatocytes as well as cholangiocytes and drainage tubules [41]. A byproduct of this secondary proliferation pathway is the development of a ductular reaction (DR). This is a reactive lesion at the portal vein interface consisting of small bile ducts and a complex of stroma and inflammatory cells [38]. Since ductal epithelium can express profibrogenic and chemotactic proteins, the latter being able to recruit and activate inflammatory and stellate cells [38], the expansion of HPCs is accompanied by the development of the HPC niche, which consists of macrophages, myofibroblasts, and matrix. These cells promote liver inflammation and fibrogenesis, which may develop into fibrosis [42]. Extensive fibrosis can block blood flow through the liver, leading to cirrhosis and hepatocellular carcinoma (HCC) (Fig. 2). Noteworthy, although HCC typically arises in the background of liver cirrhosis, about 20% of cases may develop in a non-cirrhotic liver, suggesting multiple mechanisms of hepatocarcinogenesis [43]. Therefore, understanding the molecular mechanisms required to restore adequate regenerative capacity in a chronically damaged liver is a key challenge of great clinical importance. Several pro-regenerative signaling pathways have also been found to mediate the repair of an abnormal liver [44]; among them, the Hippo signaling pathway appears to play a key role.

### The Hippo signaling pathway

**Fig. 3:**
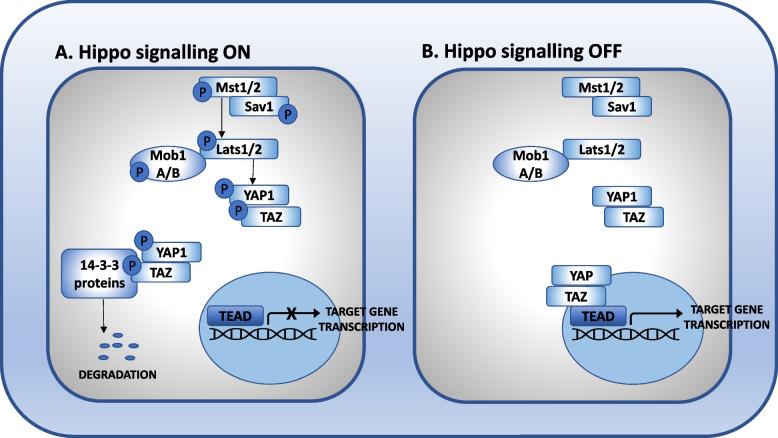
Schematic overview of the Hippo signaling pathway. **A** “Hippo signaling ON”: activation of the Hippo pathway leads to inactivating phosphorylation and cytoplasmic retention of the co-transcriptional activators YAP1 and TAZ. Specifically, activation of mammalian sterile 20-like protein kinases 1 and 2 (MST1 and MST2) activates large tumor suppressor kinases 1 and 2 (LATS1 and 2) in partnership with their scaffold molecule Salvador Family WW Domain Containing Protein 1 (SAV1). LATS1 and 2 and their partners, MOB kinase activators 1A and B (MOB1A and MOB1B), in turn phosphorylate the co-transcriptional activators YAP1 or TAZ at several serine residues causing their inactivation. When phosphorylated at specific amino acid residues, YAP and TAZ bind the scaffold molecule 14-3-3 and are eventually transported to the proteasome for degradation. **B** “Hippo signaling OFF”: when the activity of the Hippo pathway is reduced, the unphosphorylated YAP and TAZ migrate to the nucleus and associate with various DNA-binding proteins, e.g., TEA domain proteins (TEAD), which control transcription of target genes

The Hippo signaling pathway (Fig. 3), a tumor suppressor in mammals, controls organ size via regulating cellular proliferation, survival, and differentiation [45, 46]. This signaling pathway, originally described in the fruit fly Drosophila melanogaster, is named after the Hippo serine/threonine kinases (STK3/MST2 and STK4/MST1 in mammals), whose inactivation leads to organ enlargement through excessive proliferation and decreased apoptosis [47, 48]. The Hippo pathway activation results in the inactivating phosphorylation and cytoplasmic retention of the co-transcriptional activator Yes-associated protein 1 (YAP 1) or its paralog, WW domain containing transcription regulator 1 (WWTR1/TAZ). More detailed analysis revealed that mammalian sterile 20-like protein kinases 1 and 2 (MST1 and MST2) activate large tumor suppressor kinases 1 and 2 (LATS1 and 2) in partnership with their scaffold molecule Salvador family WW domain containing protein 1 (SAV1). LATS1 and 2 and their partners, MOB kinase activators 1A and B (MOB1A and MOB1B), in turn phosphorylate the co-transcriptional activators YAP1 or WWTR1/TAZ at several serine residues causing their inactivation [48]. The main relevant residues that keep YAP 1 and WWTR1/TAZ inhibited are serine (S)127 and S381 and S89 and S311, respectively [49]. When YAP is phosphorylated at these amino acid residues, it binds the scaffold molecule 14-3-3 and is eventually transported to the proteasome for degradation [49, 50]. Upon loss of Hippo function, the unphosphorylated YAP migrates to the nucleus and associates with various DNA-binding proteins, such as TEA-domain proteins (TEAD), which control gene transcription [49, 50]. TAZ is slightly smaller but has similar regulatory and activity sites [51, 52]. Several genes important for liver growth and regeneration, such as *connective tissue growth factor 28 (Ctgf 28), Jagged 1 (Jag1)* [53, 54] and *Notch receptor 2 (Notch2)*, are direct targets of Hippo signaling. Thus, increased expression of YAP/TAZ in rodent livers leads to hepatomegaly and, if persistent, to the development of HCC [48, 55–57]. Accordingly, the genomic region containing *Yap* has been amplified in breast and liver cancers [58, 59]. Of note, YAP-induced hepatomegaly is reversible, such that restoration of endogenous YAP levels leads to a rapid decrease in liver size and normalization of parenchymal architecture. Therefore, the Hippo signaling pathway appears to play an important role in maintaining liver size as part of the “hepatostat” control system that ensures adequate liver mass for the performance of organ-specific homeostatic functions. Overall, Hippo signaling is thought to be active during homeostasis, resulting in high inactivating phosphorylation of YAP/TAZ and inhibition of nuclear translocation. The Hippo signaling pathway is therefore considered tumor suppressive, as loss of Hippo signaling leads to accumulation of YAP, which translocates to the nucleus, where it triggers activation of genes that promote proliferation and prevent apoptosis [48]. Interestingly, several chronic liver diseases [60–62] characterized by impaired regenerative response are associated with YAP accumulation, although its role is currently controversial. During the development of these diseases, the expression of YAP has been found to increase in various liver cells, although its effect probably varies according to cell type. In addition, the existence of non-cell-autonomous effects of hepatocyte YAP levels has been suggested, which may affect the local microenvironment and lead to chronic inflammation, fibrosis, cirrhosis, and cancer [54].

## Hippo signaling pathway involvement during regeneration in normal liver

### Liver development and regeneration in Hippo signaling pathway knockout mice

Given the growing evidence for the importance of YAP and TAZ in controlling organ size in mammals, recent studies have examined the role of the Hippo pathway in liver regeneration. To this end, Tschuor et al. [63] knocked down *Yap1* in mouse hepatocytes prior to 2/3 PH. Knockdown reduced the expression of YAP1 during S- and M-phase peaks 36 and 48 h after surgery, respectively. Deletion of *Yap1* was associated with suppression of liver weight gain at 36 h, whereas a renewed acceleration of proliferation was observed at 48 h. Accordingly, the expression of proliferation markers, *cyclin A2/cyclin B2 (Ccna2/b2)*, and YAP1 target genes was downregulated 32 h after surgery but increased 48 h after surgery. These results suggest that YAP1 plays a key role in liver regeneration by pushing hepatocytes into the cell cycle and through S phase, while being dispensable for further cell cycle progression. Kim et al. [64] found that the Hippo signaling effector TAZ also positively modulated liver regeneration, as TAZ protein levels increased dramatically in mouse hepatocyte nuclei 24 to 72 h after 2/3 PH, with concomitant expression of cell proliferation markers. In contrast, hepatectomized *Taz* knock out (KO) mice exhibited less liver regeneration than wild-type animals (WT) in the first 3 days after surgery, with a similar liver weight to body weight ratio observed between day 5 and day 8 in both groups. The decreased liver regeneration in the livers of KO mice was related to decreased IL-6 levels compared with WT. During liver regeneration, IL-6 is mainly secreted by macrophages. The downregulation of IL-6 in KO livers was associated with a decrease in macrophage numbers, which was due to lower cytokine expression after liver injury. Therefore, TAZ was thought to regulate cytokine expression during liver regeneration, facilitating infiltration and activation of macrophages, which in turn trigger the proliferation response by releasing IL-6. Moreover, several components of the NF-kB, Janus kinase inhibitor (Jak)-Stat3, extracellular signal-regulated kinase (ERK), and protein kinase B (Akt/PKB) signaling pathways were downregulated in KO mice after 2/3 PH, indicating TAZ-dependent activation. On the other hand, KO mice showed increased apoptosis and periductal fibrogenesis after surgery, possibly due to the release of fibrogenic factors by HSCs after their activation by apoptotic bodies. Based on these data, it has been suggested that TAZ stimulates liver regeneration after liver injury through IL-6-induced hepatocyte proliferation and inhibition of cell death [64]. While these studies analyzed the effects of the Hippo pathway on liver regeneration by knocking down *Yap* or *Taz* in mouse liver, Lu et al. [65] investigated the involvement of the Hippo pathway in liver development and regeneration by conditional *Yap*/*Taz* double KO mice. These animals carried the *Yap* and *Taz* gene deletions in both hepatocytes and biliary epithelial cells (BECs). The data obtained showed that YAP and TAZ are dispensable for achieving proper organ size during normal development or in unstressed adult livers. Although KO livers were larger than their WT counterparts, this was not a direct effect of *Yap/Taz* loss, but it was rather related to liver necrosis and compensatory hepatocyte proliferation induced by toxic bile acid accumulation due to impaired BEC development. These results are consistent with those previously obtained by Lee et al. and Yi et al. [66, 67] in *Lats1* and *Lats 2* double KO mice. They found that during and after liver development, YAP /TAZ activation induced by the loss of Lats1/2 was required to force the conversion of hepatoblasts or hepatocytes into immature BECs and promote BEC proliferation [66, 67]. Accordingly, induction of Hippo coactivators was associated with upregulation of TGF-β, a known mediator of immature BEC expansion, and suppression of the hepatocyte signature gene *hepatocyte nuclear factor-1 alpha (Hnf4α)* [66]. Consistent with this, the protein level of YAP in hepatocytes decreased during maturation, and its nuclear staining diminished in a LATS1/2-dependent manner, while it was maintained at an intermediate level in BECs [67]. Since YAP is a negative modulator of hepatocyte signature genes, its inactivation was required to allow the acquisition of a mature phenotype [68]. On the other hand, Lu et al. [65] reported that activation of YAP /TAZ was necessary for adequate liver regeneration after 2/3 PH. Although the loss of both coactivators allowed proliferation of hepatocytes after surgery, it was not as efficient as in the livers of WT. Whether this defective regeneration is a direct consequence of YAP/TAZ inactivation in hepatocytes or is an indirect consequence related to defects in BECs has not been clarified in this study. However, the ability of KO livers to elicit a regeneration response suggests the possible involvement of other signaling pathways, such as those related to the activation of growth factors that may partially compensate for the loss of YAP/TAZ. Interestingly, in contrast to the results of Tschuor et al. [63] and Kim et al. [64], Lu et al. [65] observed only mild impairment of liver regeneration at the early time points after 2/3 PH, whereas major defects were observed between 7 and 14 days after surgery. This discrepancy was attributed by Tschuor et al. to the fact that the Yap/Taz double KO mice exhibited bile acid-mediated parenchymal injury, inflammation, and hepatomegaly. Thus, on the one hand, the increased basal liver proliferation may have masked the early regenerative defect, and on the other hand, the parenchymal injury may have exaggerated the regenerative deficiency over time [63]. It has also been suggested that these ambiguous results could be explained by the different knockout method [63, 69]. While in the studies of Lu [65] and Kim [64], embryonic deletion of *Yap/Taz* and *Taz*, respectively, was achieved by a liver-specific Alb-Cre driver, Tschour et al. [63] induced *Yap* deletion in adult hepatocytes by hepatocyte-specific small interfering RNAs. Since Alb-Cre becomes active in hepatoblasts that can differentiate into hepatocytes and BECs [69], the major defects observed in Alb-Cre *Yap/Taz* regenerating livers might depend on primary defects occurring during liver development and/or in mature hepatocytes or BECs, as suggested by Verboven et al. [69]. On the other hand, the ability of YAP and TAZ to compensate for each other could explain the weaker phenotypes observed in single mutants [69].

**Fig. 4:**
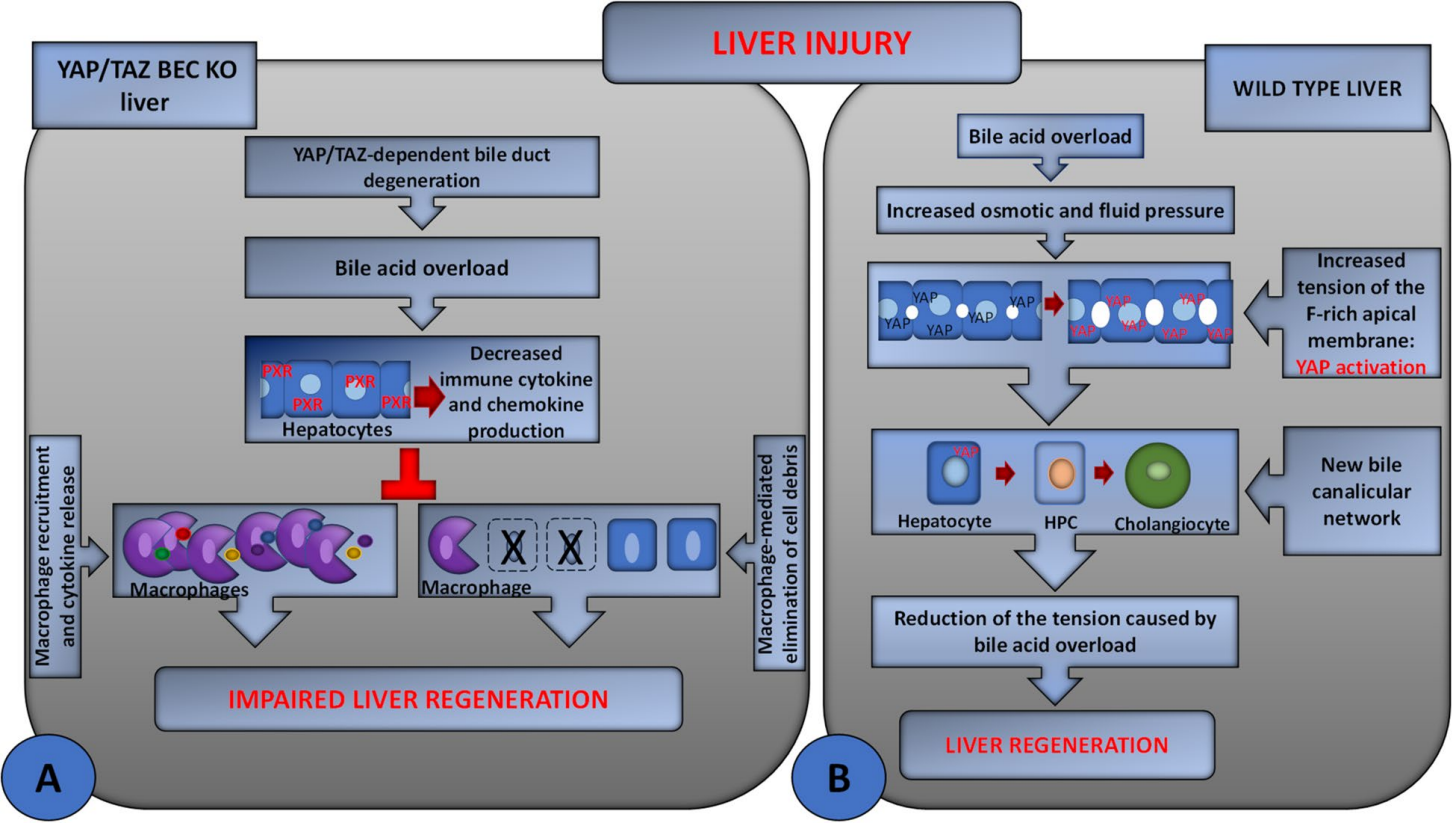
Schematic representation of the role of YAP/TAZ in liver regeneration after acute tissue injury. **A** In YAP /TAZ BEC-KO liver severe degeneration of bile ducts causes cholestasis and has secondary effects on hepatocytes and macrophages, resulting in impaired liver regeneration. More specifically, bile acid overload is responsible for PXR-mediated suppression of cytokine production in hepatocytes, which impairs phagocytic macrophage recruitment and activation. Decreased macrophage function impairs the tissue regeneration process by reducing the clearance of cellular debris from the injury site. **B** In WILD TYPE liver, injury (2/3 PH)-associated bile acid overload increases osmotic and fluid pressures in the liver, resulting in higher tension of the apical membrane of hepatocytes that form the bile canalicular network. Therefore, YAP, which is localized in the F-actin-rich region of the apical membrane of hepatocytes, is activated. YAP /TAZ positively modulate liver regeneration by promoting the trans-differentiation of hepatocytes to a BEC phenotype thus contributing to liver regeneration through the formation of new bile ducts. YAP: black, inactive; red, active

Although the requirement of YAP /TAZ for the proliferation of BECs as well as for the trans-differentiation of hepatocytes to BECs is well established [70–73], the actual contribution of YAP/TAZ to liver regeneration was only recently clarified by Verboven et al. [69] (Fig. 4 A). In this study, the function of YAP/TAZ was analyzed during liver regeneration after carbon tetrachloride (CCl4)-induced liver injury in mice with *Yap/Taz* double deletion in embryonic hepatoblasts of mice with embryonic and in mice with targeted *Yap/Taz* double deletion in adult hepatocytes or BECs. The data obtained showed that livers with *Yap/Taz* double deletion in embryonic hepatoblasts had a normal size with normal hepatocyte density and proliferation, but few and largely disorganized bile ducts and increased serum levels of transaminases indicating hepatocyte damage. Thus, YAP/TAZ are dispensable for embryonic hepatocyte proliferation and liver growth but essential for bile duct development. After CCl_4_ administration, an area of injury characterized by apoptotic hepatocytes was observed; the injury persisted also at 96 h when WT animals recovered their liver architecture. Moreover, the mutant mice showed decreased hepatocyte proliferation, with only 22% of live hepatocytes positive for the proliferation marker at the peak of proliferation compared with the 52% of control animals. However, the embryonic deletion of *Yap/Taz* in hepatoblasts did not prevent the regeneration response, although it doubled its length. When the result of *Yap/Taz* deletion in adult hepatocytes and BECs was analyzed separately, it became clear that the impairment of regeneration was the result of targeted deletion of *Yap/Taz* in BECs. Although *Yap/Taz* were activated in WT hepatocytes in response to CCl4 treatment, their targeted deletion in adult hepatocytes did not significantly affect liver regeneration. In contrast, loss of *Yap/Taz* in adult bile ducts resulted in severe defects and delayed liver regeneration characterized by decreased hepatocyte proliferation and increased apoptotic cells, resembling the knockout model of embryonic hepatoblast. These effects were due to degeneration of the bile ducts of the *Yap/Taz* mutants, which caused cholestasis and delayed the recruitment of phagocytic macrophages required for the removal of cell debris from injury sites. Macrophages from *Yap/Taz* BEC-KO mice responded only partially to toxic injury, inducing genes for cell proliferation but not activating those responsible for immune cell migration, cell debris removal, and polarization to restorative macrophages (M2 phenotype) [74]. Depletion of macrophages prior to CCl4 treatment in WT mice resulted in a phenocopy of the regenerative defects observed in *Yap/Taz* BEC-KO livers [63, 69]. From a mechanicistic point of view, bile acid overload activates the pregnane X receptor (PXR) in hepatocytes, which may be responsible for the suppression of hepatic cytokine expression and macrophage activity in *Yap/Taz* BEC-KO mice after CCl4 treatment, in agreement with other studies [75, 76].

From these data, activation of YAP /TAZ during development and in the adult liver promotes engagement and proliferation of immature biliary epithelial cells while maintaining hepatocytes in a quiescent state. Nevertheless, deletion of Yap or Taz or double deletion of Yap/Taz impairs the regenerative response of the liver after tissue injury. Interestingly, the severe defects observed in hepatectomized double Yap/Taz KO mice are dependent on the loss of Yap/Taz in adult BECs associated with severe cholestasis. Thus, bile acid overload activates PXR in hepatocytes, causing suppression of cytokine production required for recruitment and activation of phagocytic macrophages. Reduced macrophage function affects the removal of cellular debris from the injury site, resulting in impaired liver regenerative response.

### YAP activation by bile canaliculi remodeling during liver regeneration

Recently, Meyer et al. [77] linked the activation of YAP to the remodeling of the bile canalicular network during liver regeneration (Fig. 4 B). This represents a mechanosensory system in mouse hepatocytes that responds to bile acid overload caused by tissue resection. It is known that after surgery, the liver must transport the entire bile salt pool through a reduced network of tubules, resulting in increased bile salt influx per liver weight [78]. Using digital tissue reconstruction and quantitative image analysis, Meyer et al. observed that bile salt overload in hepatectomized mice caused increased osmotic and fluid pressure, fluid influx, and dilatation of the apical membrane of hepatocytes that forms the bile canalicular network. The inactivated YAP was found in the F-actin-rich region of the apical membrane of hepatocytes, an ideal localization to detect changes in bile fluid dynamics and the dilation of the bile canalicular network by conformational changes of the actin cytoskeleton. After 2/3 PH, bile acid overload increased apical membrane tension, which was sensed by F-actin. These changes determined the activation of YAP and promoted its nuclear translocation, which was dependent on the integrity of the actin cytoskeleton. This mechanism was mathematically predicted to tolerate moderate fluctuations of bile acid in tissue homeostasis but to activate YAP in response to sustained bile acid overload, such as after 2/3 PH, to promote the liver’s regenerative response. Activation of YAP promotes the dedifferentiation of hepatocyte into HPCs that can be trans-differentiated into BECs. The generation of new BECs can result in the constitution of a new bile canalicular network throughout the central vein-portal vein axis during liver regeneration, alleviating the tension associated to bile acid overload [77, 79].

In this study, the bile canalicular network was identified as a mechano-sensory system in mouse hepatocytes. After 2/3 PH, it was proposed that YAP is the effector by which the bile canalicular network activated by bile acid overload associated with liver resection promotes liver regeneration.

### The involvement of YAP in the mitogenic effect of 5-HT, BET, and Brg-1 in the liver

The crucial role of the Hippo pathway in liver regeneration has been confirmed in several studies. Fang et al. [80] demonstrated that activation of YAP is involved in promoting mouse liver regeneration by 5-hydroxytryptamine (5-HT) after 2/3 PH [81–84]. As a consequence, TPH1 knockout mice (lacking 5-HT) showed a decrease in the liver regeneration response associated with lower YAP expression and more severe tissue damage compared to their WT counterpart. In vitro experiments showed that the induction of YAP was partly due to activation of phospho-ERK. Liu et al. [85] confirmed the crucial role of YAP in liver regeneration and found that the YAP/TAZ-Notch receptor 1 (Notch1)-Notch intracellular domain (NICD) axis is critically involved in liver regeneration promoted by inhibition of bromodomain and extraterminal (BET) proteins. These proteins are a family of transcriptional regulators [86] that have recently attracted the interest of the scientific community because their inhibitors could be used to treat inflammatory diseases, metabolic disorders, cancer, and autoimmune diseases [87–90]. Based on these results and a recent study describing a key role for the protein BET during liver regeneration in a zebrafish model [91], Liu et al. [85] analyzed the effect of the BET inhibitor (S)-tert-butyl2-(4-(4-chlorophenyl)-2,3,9-trimethyl-6H-thieno[3,2-f][1,2,4]triazolo[4,3 a][1,4]diazepin-6-yl)acetate (JQ1) on liver regeneration after 2/3 PH in mice. The data obtained showed that inhibition of the BET proteins prevented liver regeneration by inhibiting the YAP/TAZ-Notch1-NICD axis. The Notch signaling pathway is a highly conserved signal transduction mechanism essential for normal embryonic development, cell proliferation, specification, and differentiation in various organisms, including mammals [92, 93]. In the liver, this signaling pathway is activated under physiological conditions by the binding of Jagged1 to the Notch1 receptor, which ultimately leads to the γ-secretase-mediated release of the Notch protein intracellular domain (NICD). NICD nuclear translocation, then, leads to the transcription of Notch target genes [94]. In the study by Liu et al. [85], JQ1-mediated inhibition of liver regeneration after 2/3 PH was associated with suppression of YAP/TAZ expression, resulting in a remarkable reduction in Jagged1 and Notch1 expression. Therefore, decreased NICD nuclear translocation and downregulation of Notch target genes involved in liver cell proliferation and differentiation were observed [95, 96]. In the same study, in vitro analysis on immortalized normal mouse hepatocytes demonstrated that the Notch signaling pathway can be directly affected by regulating the expression of YAP. Accordingly, protein levels associated with the Notch pathway increased in a mouse model that carried liver-specific Yap overexpression, and this was associated with rescue of JQ1-mediated suppression of liver regeneration. These results argue for an important role of the YAP /TAZ-Notch1-NICD axis in JQ1-dependent regulation of hepatocyte regeneration response. Nevertheless, indirect modulation of Notch1 expression by JQ1 cannot be excluded.

Recently, Gong et al. [97] analyzed the role of the Hippo pathway in liver-specific *Brahma-related gene 1 (Brg1)*-deleted (*Brg1* KO) mice after 2/3 PH. Brg1 is the core component of the SWItch/Sucrose Non-Fermentable (SWI/SNF) chromatin remodeling complex in mammals, which has ATPase activity that enables Brg1-mediated nucleosome mobilization leading to regulation of gene expression [98]. ATPase-independent transcriptional regulation by Brg1 has also been reported [99]. Several studies have shown that this protein is able to modify the expression of several genes involved in the pathogenesis of various human diseases and cellular stress responses [100–103]. In their study [97], Gong et al. found that the absence of Brg1 in mouse embryos was associated with liver cell growth disorders and a marked decrease in miR-187-5p expression, which significantly delayed liver regeneration. Interestingly, *Lats1* was identified as a target gene of miR-187-5p. Decreased expression of miR-187-5p during liver regeneration in liver-specific *Brg1* KO mice led to activation of the Hippo pathway via increased expression of *Lats1*, resulting in decreased levels of cell cycle-related proteins and delayed liver regeneration. The impairment of liver regeneration was rescued by transfusion of miR-187-5p analogs, which led to inactivation of the Hippo pathway and increases in cell cycle proteins.

The data presented here demonstrate that YAP plays a critical role in mediating the liver regeneration promoting effect of various factors after 2/3 PH. Namely, it has been shown that (i) the 5-HT-mediated promotion of liver regeneration after 2/3 PH is associated with the induction of YAP expression in hepatocytes, which may depend on phospho-ErK activation; (ii) inhibition of BET proteins prevents liver regeneration after 2/3 PH in mice and in culture cells by inhibiting the YAP /TAZ-Notch1-NICD axis; and (iii) Brg1 mediates liver regeneration after 2/3 PH by reducing the expression of miR-187-5p, a negative regulator of LATS1, thereby inactivating growth-suppressive Hippo signaling and promoting cell cycle progression.

## Involvement of Hippo pathway in tissue repair in abnormal liver

### The role of YAP and TAZ in nonalcoholic fatty liver disease (NAFLD)

**Fig. 5:**
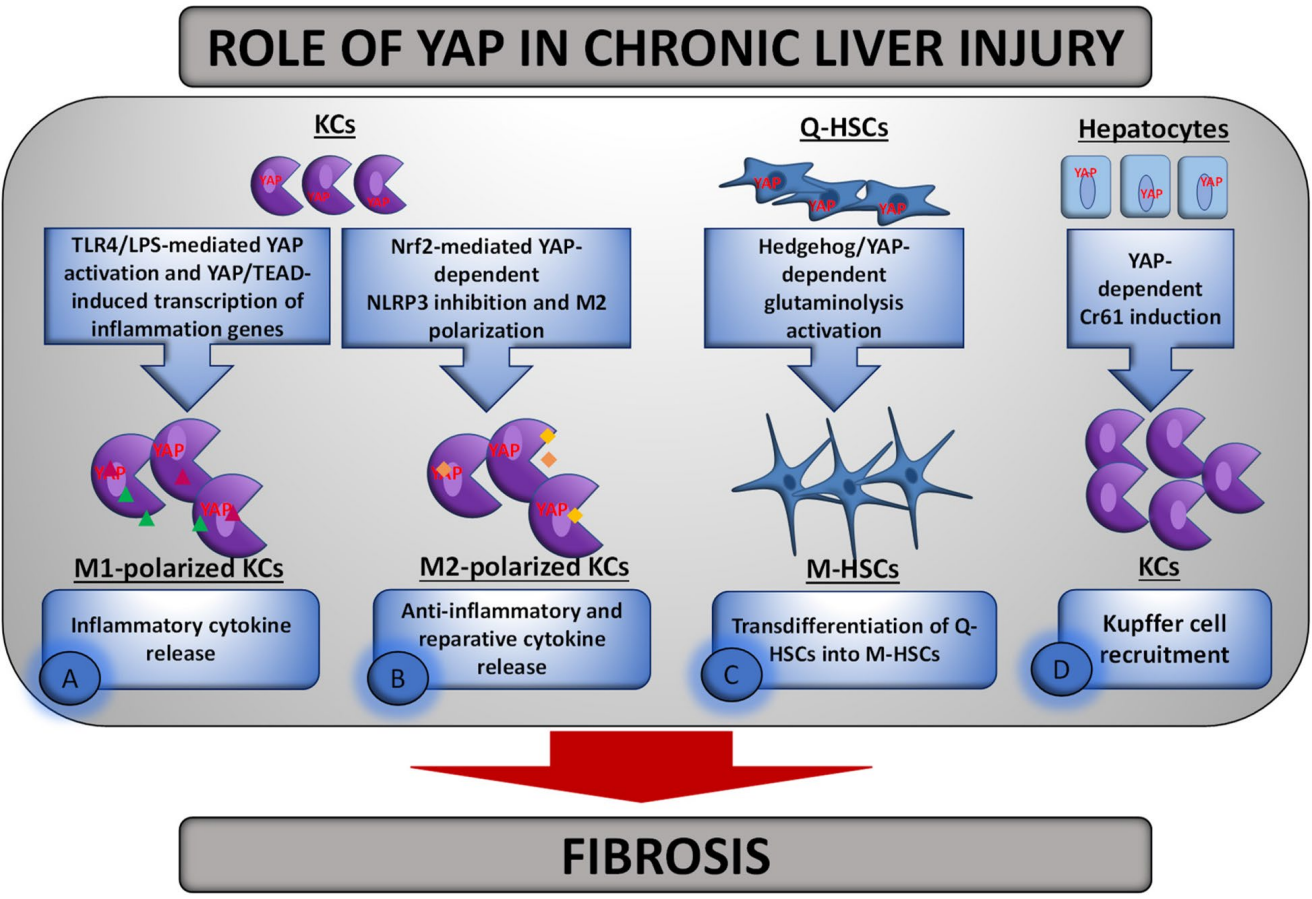
Schematic representation of the role of YAP/TAZ in chronic injured liver. YAP activation in chronically injured liver leads to a wound healing response characterized by (*A*) YAP/TEAD mediated transcriptional activation of inflammatory genes in KCs leading to the release of inflammatory cytokines; (*B*) inhibition of NLRP3 inflammasome and induction of M2 polarization in KCs, accompanied by the release of anti-inflammatory and reparative cytokines; (*C*) trans-differentiation of Q-HSCs to M-HSCs, which is due to Hedgehog/ YAP-mediated stimulation of glutaminolysis in Q-HSCs; and (*D*) induction of Cyr61 expression in hepatocytes, which promotes macrophage recruitment

Non-alcoholic fatty liver disease (NAFLD) ranges from simple non-alcoholic fatty liver to injury and inflammation characteristic of non-alcoholic steatohepatitis (NASH), which in turn can develop into fibrosis. Fibrosis can progress to cirrhosis and HCC [104]. Hepatomegaly is a common feature in NAFLD patients, but progressive liver fibrosis rarely occurs [105]. The severity of liver fibrosis predicts liver-related morbidity and mortality [106] and correlates strongly with the accumulation of cells with liver progenitor abilities known as reactive-appearing ductular liver cells (RDCs), both processes being more pronounced in NASH than in less severe liver injury (i.e., non-NASH /single steatosis) [38]. Interestingly, experimental YAP activation in mouse hepatocytes was found to trigger an accumulation of stem-like cells in the liver resembling RDCs [54]. These YAP-positive RDCs are able to engraft into the injured liver after transplantation and eventually repopulate it with healthy, mature hepatocytes, thereby rescuing recipients from liver failure [54]. In 2015, Machado et al. [60] found an increased number of YAP-positive RDCs in both NASH patients and NASH mouse models. The accumulation of YAP-positive RDCs correlated with the severity of hepatocyte injury and the increased release of Sonic hedgehog (Shh) ligands from dying hepatocytes. Once released into the microenvironment, these ligands stimulated the growth of cells involved in liver repair, including RDCs and liver myofibroblasts, which in turn released Shh ligands and other fibrogenic factors [60]. Therefore, the accumulation of RDCs showing Shh-mediated induction of YAP was warranted by autocrine/paracrine signaling [60, 106]. Although the role of YAP in NASH remains to be elucidated, these results have supported the hypothesis that YAP is deregulated in NAFLD patients who are at high risk for cirrhosis. NASH is one of the most common causes of liver disease worldwide and is currently the second most common indication for liver transplantation in the USA [107]. However, because few livers are available for transplantation, it is extremely urgent to identify new pharmacological targets that can be used in patients at high risk for NASH (i.e., obese individuals). In addition, a comprehensive understanding of the pathophysiology of the disease is desirable. Recently, Song et al. [108] linked the development of NASH to YAP-dependent regulation of KCs (Fig. 5 A). They found that YAP levels were greatly increased in the livers of mice fed a high-fat diet (HFD) which developed NAFLD, with KCs being the major cell type with high YAP-expression. These cells promoted liver inflammation through YAP-mediated production of pro-inflammatory cytokines. Accordingly, deletion of the *Yap* gene in KCs or pharmacological inhibition of the protein attenuated liver inflammation in NAFLD mice. Transcriptional activation of YAP in KCs has been associated with induction of gut-derived endotoxin/lipopolysaccharide (LPS)/Toll-like receptor 4 (TLR4) signaling [109]. Deletion of TLR4 in KCs or pharmacological inhibition of YAP attenuated LPS-induced YAP and pro-inflammatory cytokine expression, respectively, without altering the degree of steatosis. Of note, transcriptional activation of YAP by LPS in KCs determined the production of pro-inflammatory cytokines (including monocyte chemoattractant protein-1, MCP-1, TNF-α, and IL-6) because of the association of YAP with the TEAD-binding motif in the promoter region of inflammatory cytokine genes. It is well known that progression of NAFLD is characterized by M1/M2 polarization of macrophages [110]. While M1-polarized KCs exhibit a pro-inflammatory phenotype leading to pro-inflammatory cytokine secretion, M2-polarized KCs exhibit an anti-inflammatory reparative phenotype. Consistent with this, M1-KCs represent the predominant phenotype in NASH [110, 111]. In Song study, specific deletion of *Yap* in macrophages/monocytes from HFD-fed mice resulted in a decrease in M1 and an increase in M2 markers in KCs, providing new evidence for YAP-mediated M1/M2 polarization [108]. Moreover, a positive correlation between YAP and the stage of liver fibrosis was found in human NASH tissue, confirming that YAP-dependent KC-mediated inflammation contributes to HSC activation and fibrogenesis at NASH [108]. Remarkably, the association between Hippo pathway inactivation and liver fibrosis was also noted by Wang et al. [112], who analyzed TAZ expression in human livers affected by NASH-related fibrosis and in various mouse NASH models. Of note, the positive correlation between YAP and liver fibrosis was absent in the mouse model of NAFLD by Song et al., possibly due to the lower fibrogenic efficacy of the HFD feeding protocol in mice. On the other hand, in both Song’s study and Wang’s [108, 112], inactivation of the Hippo pathway promoted fibrosis in NASH liver, without affecting the degree of steatosis, suggesting that activation of YAP in KCs may only regulate inflammatory responses and not lipid metabolism. Recently, Wang et al. [113] found a crucial link in liver macrophages between YAP activation and the expression of (erythroid-derived 2)-like 2 factor (NFE2L2, best known as Nrf2), the transcription factor considered as the “master switch” of intracellular redox homeostasis [114], during NASH development (Fig. 5 B). They demonstrated for the first time in mouse models of NASH and in patients with hepatic steatosis that liver macrophages are characterized by decreased Nrf2 expression. Accordingly, myeloid-specific Nrf2-deficient (Nrf2^M-KO^) mice showed exacerbation of hepatic steatosis and inflammation after HFD feeding compared with the chow diet group. Increased production of inflammatory cytokines resulted in increased lipid metabolism in hepatocytes of Nrf2^M-KO^ mice, both in vivo and in vitro. It was found that the protective effect of Nrf2 against NASH progression was due to its regulation of YAP-mediated NLR family pyrin domain containing 3 (NLRP3) inflammasome activity. The NLRP3 inflammasome is a multiple protein complex that plays a critical role in the host immune response to infection or sterile injury [115]. Once activated by a broad spectrum of microbial components, endogenous danger signals, and environmental irritants [115], NLRP3 mediates the activation of caspase-1, which induces the maturation of interleukin (IL)-1β and IL-18 as well as pyroptotic cell death mediated by gasdermin D [115]. The deregulated NLRP3 inflammasome is known to drive the progression of many inflammatory, metabolic, degenerative, and age-related diseases [116, 117], including NASH [118]; therefore, its activity must be tightly regulated to avoid deleterious effects.

Nrf2 regulates the inflammatory response mainly by eliminating reactive oxygen species (ROS), which are potent activators of various inflammatory pathways, although the exact molecular mechanisms are still unclear. ROS overproduction has been shown to promote MST1/2 phosphorylation [119]. Accordingly, in the study by Wang [113], Nrf2 deficiency was associated with the accumulation of ROS, which promoted phosphorylation of MST1/2, LATS1, and YAP under steatogenic conditions, leading to activation of Hippo signaling, promotion of YAP degradation, and reduction of its nuclear level. Further evidence for ROS-dependent YAP regulation is that in vitro treatment of Nrf2-deficient macrophages with antioxidants restored YAP activation by decreasing MST1/2, LATS1, and YAP phosphorylation. YAP inactivation impaired inflammasome activity in Nrf2-deficient mice under steatogenic conditions. Consequently, NLRP3 activation in bone marrow-derived macrophages (BMDMs) isolated from Nrf2^M-KO^ mice exposed to the steatogenic compound palmitic acid (PA) was significantly higher than in control cells, and this effect was counterbalanced by restoration of YAP expression in vitro. A recent study by Li et al. [120], discussed later, has shown that in macrophages activated YAP acts as a coactivator for β-catenin, which in turn regulates its target gene *X-box binding protein 1 (Xbp1)*, resulting in decreased NLRP3/caspase-1 activity. Moreover, the YAP-β-catenin interaction was found to play a key role in triggering the anti-inflammatory M2 macrophage phenotype. On this basis, the expression of XBP1 and β-catenin in the PA-treated BMDMs of Nrf2^M-KO^ and WT mice was analyzed by Wang et al. [113]. The data obtained showed that Nrf2 deficiency increased Xbp1 expression but downregulated β-catenin protein levels, indicating higher NLRP3 activity [113]. Restoration of YAP expression by lysophosphatidic acid (LPA) treatment attenuated steatohepatitis in Nrf2^M-KO^ mice with NASH and was associated with decreased NLRP3 activation in macrophages isolated from mouse livers. Taken together, these data suggest that Nrf2 deficiency exacerbates inflammation in NASH livers by promoting Hippo signaling in a ROS-dependent manner, which in turn regulates NLRP3 activation in a YAP /β-catenin/XBP1-dependent manner. Thus, Nrf2 exerts a protective role in macrophages against NASH progression by modulating YAP-mediated NLRP3 inflammasome activity. Further evidence for this finding is that overexpression of Nrf2 in WT mice exposed to HFD feeding resulted in significant improvement in liver inflammation and steatosis compared to the control group [113].

In conclusion, conflicting results have been reported on the role of the Hippo pathway in modulating innate immune responses in NASH livers. The studies presented here have shown that inactivation of the Hippo pathway in NASH livers contributes to fibrogenesis through YAP-dependent transcriptional activation of macrophage inflammatory genes, leading to M1/M2 polarization. On the other hand, Wang et al. found a protective role for YAP activation during NASH. Specifically, they have shown that in NASH livers, macrophage Nrf2 regulates ROS-mediated stabilization of YAP, which in turn inhibits NLRP3 inflammasome activation, leading to amelioration of steatohepatitis symptoms. Accordingly, Nrf2 deficiency exacerbates hepatic steatosis and inflammation in NASH livers. These results indicate that induction of YAP by Nrf2 is a protective factor during NASH that attenuates liver inflammation.

### The role of YAP /TAZ in liver fibrosis

Liver fibrosis is one of the most common consequences of chronic liver disease. It can be classified as a wound healing response to chronic liver injury, which may be caused by various factors such as alcohol abuse, drugs, hepatitis virus infection, autoimmune disease, biliary obstruction, or non-alcoholic steatohepatitis. Once developed, liver fibrosis is characterized by excessive scarring due to overproduction and deposition of extracellular matrix (ECM) components caused by increased synthesis or decreased degradation of ECM components, or both [121, 122]. Liver function is significantly impaired during fibrosis, with architectural and functional changes triggering a positive feedback loop that further amplifies the fibrogenic process and leads to the progression of liver cirrhosis and organ failure [122]. It has been shown that YAP/TAZ play an essential role in liver fibrosis. Accordingly, the two Hippo transcriptional coactivators were found to (i) be involved in the transcriptional regulation of many pro-fibrogenic genes [54, 123–125]; (ii) regulate the biogenesis of liver fibrosis-related miRNAs [126, 127]; (iii) promote hepatic fibrosis in mouse liver by activating HSCs in response to chronic damage [128]; (iv) accumulate in myofibroblasts and HSCs of human and mouse fibrotic livers [128, 129]; (v) increase their own expression in parallel with bile duct proliferation and fibrosis [60, 61, 130]; and (vi) mediate the antifibrogenic effect of omega-3 polyunsaturated fatty acids (ω-3 PUFAs) in mouse liver [131]. A crucial event in the pathogenic sequence of liver fibrosis is the activation of HSCs, an important cell type responsible for the increased synthesis of ECM proteins. During liver injury, these cells differentiate into a myofibroblastic phenotype characterized by overexpression of α-smooth muscle actin (α-SMA), collagen type I and III, fibronectin, etc. [132]. It is well known that trans-differentiation of quiescent HSCs (Q-HSCs) into myofibroblastic HSCs (M-HSCs) and maintenance of the M-HSC phenotype requires liver injury-induced activation of the Hedgehog signaling pathway [133]. Like the Hedgehog pathway, YAP is relatively inactive in healthy livers but is dramatically activated in HSCs upon tissue damage [134]. The Hedgehog signaling pathway has been shown to control the activity of YAP in HSCs, which is required to initiate the trans-differentiation of Q-HSCs into M-HSCs [134]. In terms of their bioenergetics and biosynthetic requirements, M-HSCs resemble highly proliferative cancer cells. Metabolic reprogramming required for cancer cell growth has been shown to critically depend on glutaminolysis [135]. On this basis, Du et al. [136] analyzed the involvement of glutaminolysis in the metabolic reprogramming leading to trans-differentiation of Q-HSCs to M-HSCs and investigated whether this process is controlled by Hedgehog signal transduction-mediated regulation of YAP. The data obtained confirmed that stimulation of glutaminolysis is required for M-HSC growth and for the acquisition and maintenance of the myofibroblastic phenotype (Fig. 5 C). Indeed, in vitro experiments with primary mouse HSCs and rat M-HSCs showed that these processes are mediated by glutamine catabolism leading to the production of alpha-ketoglutarate (α- KG). Since this metabolite is known to increase the activity of ATP-generating metabolic pathways such as the tricaboxylic acid cycle [137], it represents the ultimate effector to meet the high bioenergetic and biosynthetic requirements of M-HSCs. Consequently, the expression of *Glutaminase1 (Gls1)*, the gene encoding the first enzyme of the catabolic pathway that converts glutamine to glutamate, was upregulated in M-HSCs. Moreover, deprivation of glutamine or pharmacological inhibition of *Gls1* inhibited the growth of M-HSCs and prevented HSCs from developing the myofibroblastic phenotype. In support of the in vitro data, an in vivo model of CCl4-mediated liver injury in mice showed that induction of glutaminolysis was important for the accumulation of M-HSCs in damaged livers [136]. To determine whether this is also the case in chronic fibrotic livers, *Gls1* expression was analyzed: (i) in mouse and human primary HSCs and rat myofibroblastic HSC cultures, (ii) in mouse models of liver fibrosis (induced by methionine-choline deficient diet or CCl4 administration), (iii) in transcriptomic data derived from microarray analysis of over 70 NASH patients with varying degrees of liver fibrosis [138], and (iv) in liver biopsies from healthy human livers and patients with histologically graded fibrosis. The data obtained suggest that glutaminolysis is a conserved driver of M-HSC accumulation during liver fibrosis, as shown by the positive correlation between upregulation of *Gls1* and the degree of liver fibrosis [136]. Experiments using Hedgehog signaling conditional KO mice have shown that trans-differentiation of HSCs is controlled by Hedgehog-mediated activation of YAP [136]. Consistent with this, silencing of *Yap* in rat M-HSCs was associated with suppression of *Gls1* expression. Moreover, pharmacological inhibition of YAP in M-HSCs resulted in a dramatic decrease in mitochondrial respiration and cell growth, which was reversed by supplementing the culture medium with a cell-permeable analog of α-KG. All these data have demonstrated a link between glutaminolysis and Hedgehog-YAP signaling and provide a mechanism to explain why genetic approaches associated with direct disruption of Hedgehog-YAP signaling inhibit M-HSC accumulation [134, 139]. As Hedgehog and YAP signaling are key pathways involved in controlling energy consumption and maintaining myofibroblastic properties, recently Bruschi et al. [140] investigated whether the I148M variant of the PNPLA3 gene could promote TGF-β- and leptin-induced liver fibrosis in primary HSC cells by impairing Hedgehog and YAP signaling. PNPLA3 or adiponutrin is the most closely related to ATGL/PNPLA2 member of the patatin-like phospholipase domain-containing (PNPLA) family [141]. Several studies have shown an association between liver fibrosis and PNPLA3 expression in HSC cells [142, 143] and activated HSCs carrying the I148M variant of the PNPLA3 gene have been found to exhibit enhanced pro-inflammatory and pro-fibrogenic properties [144]. The data obtained in the study by Bruschi [140] showed that TGF-β treatment rapidly upregulated the expression of profibrogenic genes, namely *Collagen Type I Alpha 1 Chain (COL1α1), Carnitine palmitoyltransferase 1A, (Cpt-1),* and *Fatty acid synthase (Fasn*), YAP /hedgehog target genes, namely *Amphiregulin* (*Areg), Survivin, Snail Family Transcriptional Repressor 1 (Snail), Forkhead Box M1 (Foxf1), Cyclin D1 (Ccnd1), and Vimentin*, and gene and protein expression of PNPLA3 in primary HSCs. Overexpression of PNPLA3 I148M in HSCs (I148M HSCs) resulted in enhanced anaerobic glycolysis and YAP and Hedgehog signaling activation compared with WT cells. In addition, exposure of I148M HSCs to TGF-β and leptin further enhanced the expression of YAP. Conversely, pharmacological inhibition of YAP by veteporfirin (VP) strongly abrogated YAP-mediated gene expression in both untreated and TGF-β/leptin-treated I148M-HSCs. It has been reported that activation of peroxisome proliferator receptor-γ (PPARγ) signaling in HSCs is accompanied by loss of their myofibroblastic properties, which are mediated by Hedgehog signaling-dependent YAP activation [129, 134, 145]. Accordingly, Bruschi et al. found that I148M HSCs exhibit specifically decreased PPARγ signaling [144] and that treatment of these cells with the synthetic PPARγ agonist rosiglitazone in combination with VP greatly reduces YAP transcriptional activity.

Recently, Mooring et al. [146] have sought to better characterize the contribution of YAP to the progression and reversal of liver fibrosis and the underlying mechanisms. They found that transgenic expression of *Yap/Taz* in mouse hepatocytes correlates with activation of ligands that promote fibrosis (COL1A1, tissue inhibitor of metalloproteinases 1 (TIMP1), platelet-derived growth factor c (PDGFc), TGFβ2) and inflammation (TNF-α, IL1β) after CCl4 injury. The positive correlation between YAP activation and fibrosis was confirmed using several mouse models of chronic liver injury [146]. In contrast, hepatocyte-specific deletion of *Yap* or *Yap/Taz* resulted in decreased myofibroblast expansion, inflammation, and fibrosis after CCl4 injury, although the degree of necrosis was similar to controls. Consistent with this, the expression of Cyr61, a known target gene of the Hippo pathway [147] that has been described as an inducer of macrophage chemoattraction in NASH [148], was decreased in liver-specific *Yap/Taz* KO mice, whereas it was upregulated in controls [146]. As a general trend, metabolic genes were more upregulated in double KO mice than in control mice, possibly reflecting the inefficient use of scarce cellular resources, while genes related to immune cell migration were less activated compared to controls. As a result, KO mice exhibited less liver inflammation and fibrosis compared with WT. In vivo experiments confirmed the key role of *Cyr61* in controlling liver fibrosis and inflammation in a YAP/TAZ-dependent manner. Moreover, a direct correlation between the levels of YAP/TAZ and Cyr61 was found in the liver tissues of patients with high-grade NASH. From these results, it appears that during liver injury YAP/TAZ/Cyr61 hepatocyte levels increase in mice and humans to determine Cyr61-dependent recruitment of macrophages that promote inflammation and fibrosis (Fig. 5 D).

In summary, these studies suggest that the Hippo signaling pathway may play a key role in the induction of liver fibrosis after liver injury. Accordingly, blocking YAP expression alleviates CCl4-induced progression of liver fibrosis in mice and promotes its regression. The pro-fibrogenic effect of YAP/TAZ was found to be related to at least two events: (i) the induction of trans-differentiation of Q-HSCs to M-HSCs and proliferation of M-HSCs, which depends on Hedgehog/YAP-mediated glutaminolysis stimulation, and (ii) the recruitment of macrophages by YAP /TAZ-mediated Cyr61 induction in hepatocytes.

### The role of YAP and TAZ in liver repair after ischemia-reperfusion injury

Hepatic ischemia-reperfusion (I/R) injury (IRI) induced by hypoxia stress is one of the major complications of liver resection, transplantation, and trauma [149, 150]. Hepatic I/R leads to an acute inflammatory response followed by hepatocellular injury mediated by ROS [151]. Liver dysfunction occurring after I/R is associated with high morbidity and mortality. In experimental models of liver IRI, the peak of hepatocellular injury usually occurs within 24 h after reperfusion, followed by liver repair and regeneration involving a complex network of signal transduction and cellular remodeling processes [152, 153]. In the early phase after I/R, HSCs release proinflammatory mediators that enhance acute liver injury [154]. Since the Hippo signaling pathway is associated with HSC activation [128], in 2018, Konishi et al. [155] investigated whether YAP and TAZ regulate HSC biology during IRI in a mouse model with partial (70%) I/R. The results showed that the pronounced activation and proliferation of HSCs after I/R were characterized by the selective activation and nuclear translocation of YAP and TAZ. Accordingly, the YAP /TAZ inactivating kinases, LATS1 and MOB1, were downregulated in HSCs after I/R, whereas YAP and TAZ target genes, *Ctgf* and *Survivin*, were upregulated. HSC expansion and simultaneous activation of YAP and TAZ were not observed in non-ischemic control livers. Accordingly, inhibition of YAP and TAZ by VP decreased HSC proliferation and expression of the YAP targets survivin [156] and cardiac ankyrin repeat protein [128] in livers after I/R. These changes were associated with a significant decrease in hepatocyte proliferation, suggesting that liver repair and regeneration responses after I/R are determined by YAP- and TAZ-dependent HSC proliferation. Recently, Liu et al. [157] investigated whether and how YAP signaling could affect hepatocyte function, innate immune response, and HSC-mediated tissue repair/fibrosis during IRI. To this end, they studied patients with orthotopic liver transplantation (OLT), a condition associated with IRI that can lead to acute and chronic rejection [158, 159], and a well-established liver IRI model in mice. The data obtained showed that high perioperative expression of YAP was associated with better preserved liver histopathology and liver cell function in OLT recipients. In addition, the parallel study in the mouse IRI model demonstrated the key role that YAP plays in hepatocellular protection and HSC-mediated fibrosis. While post ischemic livers were damaged and had low levels of YAP, a single intravenous infusion of the YAP activator LPA before the onset of ischemia resulted in hepatic cytoprotection against I/R insult. This was evidenced by decreased serum alanine transaminases (sALT), enhanced regenerative/antioxidant gene program, attenuated hepatocellular necrosis/apoptosis, and decreased innate immune responses compared with LPA-untreated post ischemic livers. In addition, LPA pre-treatment preserved liver architecture, reduced fibrosis formation during recovery, and suppressed ECM synthesis. In contrast, treatment with VP reversed YAP-mediated protection against IRI, resulting in abundant ECM synthesis, massive collagen deposition, and fibrosis development. In vitro studies performed on primary mouse hepatocyte cultures that suffered from hypoxia-reoxygenation confirmed the protective role that activation of YAP exerts on cell viability by enhancing regenerative/antioxidant gene programs. Interestingly, in Konishi’s study, YAP /TAZ was found to mediate the activation and proliferation of HSCs during IRI [155], while in Liu’s study, YAP-mediated protection of I/R-stressed hepatocytes was associated with the reduction of the release of pro-inflammatory mediators by macrophages as well as HSC activation [157]. Indeed, they found that resting HSCs, unlike hepatocytes, were insensitive to activation of YAP, which is known to promote differentiation into myofibroblasts [128], resulting in minimal liver fibrosis. On the other hand, inhibition of YAP was able to exacerbate hepatocyte injury, resulting in a strong proinflammatory response, activation of HSCs, and liver fibrogenesis. Based on these observations, Liu et al. proposed that HSC-mediated liver fibrosis requires two fibrogenic signals: a first stimulus for quiescent HSCs by I/R-induced pro-inflammatory cytokines, followed by a second stimulus mediated by activated YAP. In the case of activation of YAP by LPA before the onset of ischemia, the resulting low concentration of pro-inflammatory cytokines was ineffective in activating differentiation of HSCs into myofibroblasts, which explained the protection against liver fibrosis. Interestingly, LPA failed to protect the liver from IRI in Nrf2-deficient mice [114], first suggesting that Nrf2 signaling is required for YAP-mediated cytoprotection in IRI liver. The mechanism associated with YAP-mediated cytoprotection in IRI liver was further characterized by Li et al. [121]. They found that adoptive transfer of mesenchymal stem cells (MSCs) 24 h before hepatic IRI in mice reduced hepatocellular damage, induced M2 macrophage polarization, and decreased the inflammatory response. The protective effect of MSC treatment in ischemic liver was associated with decreased MST 1/2 and LATS 1 phosphorylation, increased nuclear YAP and β-catenin expression, and increased prostaglandin E2 (PGE2) production. Conversely, deletion of myeloid YAP or β-catenin in MSC-transferred mice exacerbated IR-induced liver inflammation, increased NLRP3/caspase-1 activity, and reduced M2 macrophage phenotype. Therefore, MSC-mediated immune regulation affected both YAP and β-catenin activity. On the one hand, MSC treatment reduced IR-induced activation of the Hippo pathway, resulting in increased YAP nuclear translocation in ischemic livers; on the other hand, increased IR-induced PGE2 release from MSCs activated the macrophage Akt, which in turn phosphorylated β-catenin at Ser552, resulting in its nuclear translocation. Using an MSC/macrophage coculture system, nuclear YAP was found to interact with β-catenin in macrophages, which in turn regulated the expression of its target gene Xbp1, resulting in decreased NLRP3/caspase-1 activity. In addition, the interaction between YAP and β-catenin was found to play a key role in shifting macrophage polarization toward the M2 phenotype, although this effect remains to be fully characterized. Consistent with this, myeloid YAP or β-catenin deficiency in MSC-treated livers reduced *Arginase 1 (Arg1)* expression in M2 macrophages, whereas proinflammatory cytokine genes increased in response to IR stress [121]. Of note, increased NLRP3 expression inhibited *Arg1* expression in M2 macrophages but enhanced *inducible nitric oxide synthase (iNOS)* expression in M1 macrophages, which was associated with increased IL-1β release after coculture with MSCs. Therefore, the data obtained demonstrate that NLRP3 exerts a central role in modulating M1/M2 macrophage polarization during YAP-β-catenin-mediated regulation.

The studies reported here have shown that activation of YAP/TAZ in IRI plays a hepatoprotective role, leading to regeneration/antioxidant gene promotion, reduced cell death, and suppression of the innate inflammatory response. This hepatoprotective effect depends on YAP induction before the onset of ischemia, whereas when pro-inflammatory cytokines are released as a result of ischemia, liver fibrosis mediated by YAP-dependent HSC regulation is the major outcome. Characterization of the molecular mechanisms associated with the immunosuppressive role of YAP during IRI has shown that (i) it acts as a coactivator of β-catenin; (ii) the YAP-β-catenin interaction plays a key role in inhibiting XBP1-mediated NLRP3 inflammasome activation; and (iii) YAP reprograms macrophage differentiation toward an M2 phenotype.

## The role of YAP in modulation of hepatocarcinogenesis

### YAP-dependent elimination of damaged hepatocytes

**Fig. 6:**
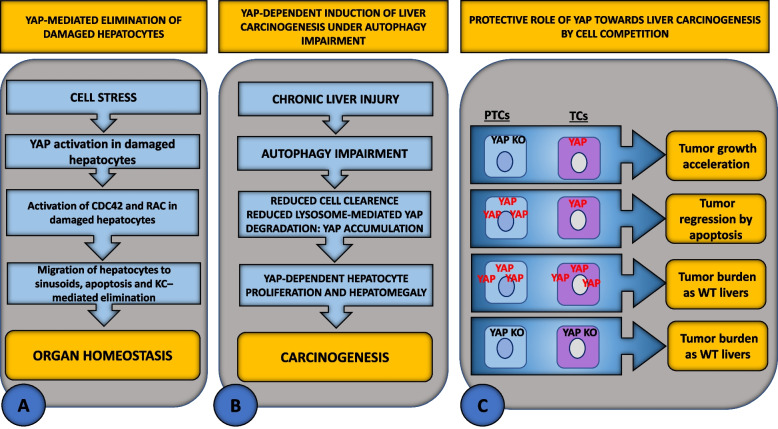
Schematic representation of YAP-mediated modulation of liver carcinogenesis. **A** Activation of YAP in damaged hepatocytes protects against liver carcinogenesis by activating the GTPases RAC and CDC42, which regulate cytoskeletal organization. Activation of CDC42 and Rac drives migration of damaged hepatocytes to sinusoids, where they are eliminated by KCs. **B** YAP acts as a trigger of liver carcinogenesis under the condition of impaired autophagy, which affects cell clearance leading to accumulation of YAP due to decreased lysosomal-mediated degradation of YAP. **C** A mechanism of tumor suppression by YAP and TAZ based on a competitive interaction between tumor cells (TCs) and their environment has been described. Deletion of Yap/Taz in peritumoral cells (PTCs) accelerates tumor growth, whereas its overexpression suppresses tumor growth and can cause tumor regression. When competition is neutralized, e.g., when PTCs and TCs simultaneously overexpress or delete Yap/Taz, the tumor burden is similar to that in WT liver. Thus, tumor cells require YAP/TAZ for survival, but only when surrounded by WT hepatocytes

Cell stress leads to senescent transformed or damaged cells [160]. Since these cells may impair tissue and organ function or lead to tumorigenesis, they must be eliminated, and their loss replaced by cell proliferation [161]. The molecular mechanisms involved in the maintenance of tissue and organ homeostasis during cellular stress are currently largely unknown. The liver is one of the most important detoxification organs; therefore, it is constantly exposed to various stresses. Given the importance of the Hippo pathway in regulating liver size and carcinogenesis by modulating cell death and proliferation, Miyamura et al. [162] analyzed the dynamics of YAP-activating hepatocytes in mice during liver injury. By transfecting mouse livers with Myc-tagged YAP wild type (WT) or YAP active mutants, they found that activation of YAP in undamaged hepatocytes leads to proliferation, whereas in damaged hepatocytes it promotes their selective elimination. In the presence of ethanol, which is capable of inducing senescence in both hepatocytes and liver endothelial sinusoidal cells (LESCs), hepatocytes expressing activated YAP migrated into hepatic sinusoids, underwent apoptosis, and were engulfed by KCs. The YAP-dependent switch from proliferation to migration/apoptosis observed in injured hepatocytes and identified by their positivity for the senescence-associated marker β-galactosidase (SA β-gal), was associated with upregulation of *Ect2* and *Fgd3 genes* in damaged cells. These genes encode guanine nucleotide exchange factors for CDC42 and Rac, two small GTP proteins of the Rho family that regulate cytoskeletal organization and cell migration [163, 164]. Therefore, upregulation of *Ect2* and *Fgd3* triggered the activation of CDC42 and Rac in hepatocytes and drove their migration to sinusoids, where they were eliminated by KCs. Based on this finding, the authors proposed that YAP maintains tissue and organ homeostasis by acting as a stress sensor that triggers the elimination of damaged cells (Fig. 6 A).

In summary, these studies indicate that YAP acts on cells depending on their status. While activation of YAP triggers a proliferation response in normal cells, in damaged cells, i.e., senescent or injured cells, it promotes their elimination by apoptosis to maintain tissue homeostasis and protect against tumorigenesis.

### YAP-dependent promotion of hepatocarcinogenesis under the condition of impaired autophagy

Autophagy is the major inducible pathway for the degradation of cellular components in eukaryotic cells via a lysosome-dependent machinery [165]. In chronically damaged livers, autophagy is usually impaired, leading to decreased clearance of cellular components and impaired mitochondrial and cellular integrity [166], possibly promoting tumor development. Although chronic liver disease is characterized by a reduction in autophagy, the relationship between loss of autophagy and carcinogenesis is still unclear [167–169]. It is known that autophagy plays a dual role in cancer. During tumor initiation, it acts as a tumor suppressor mechanism leading to the elimination of damaged organelles and ROS to ensure genomic stability and promote senescence triggered by oncogenes. However, in advanced tumors and metastases, autophagy may promote tumor cell survival under nutrient-deficient conditions and chemotherapy-induced stress [170, 171]. Although further studies are needed, the role of autophagy in the liver is primarily tumor suppressive. For example, Takamura et al. [172] found several years ago that in mice hepatocyte-specific silencing of essential genes relevant to autophagy, such as *Autophagy-related 5 (Atg5)* and *7 (Atg7)* [166, 173], stimulated hepatomegaly and the formation of adenomas but not HCCs. This effect was associated with the accumulation of the protein Sequestosome-1 (SQSTM1/also called p62), a typical autophagic substrate [174], due to impairment of autophagy. The SQSTM1 protein is able to bind and inhibit Kelch-like protein 1 (KEAP1), a negative regulator of Nrf2 that, when stabilized, acts as a pro-tumor transcription factor by determining a deleterious high antioxidant response [175]. In a recent paper, Lee et al. demonstrated that YAP contributes to the malignant transformation of hepatocytes lacking *Atg7* gene [176] (Fig. 6 B). Specifically, they found that YAP rapidly accumulated after liver-specific deletion of *Atg7* in mice and drove hepatocyte proliferation, leading to severe hepatomegaly and the development of HCC. Accumulation of YAP was dependent on a general deficiency in the autophagy pathway, resulting in reduced lysosome-mediated degradation of YAP. Remarkably, the accumulation of YAP preceded the accumulation of SQSTM1, which occurred several weeks later [176]. Interestingly, the liver-specific double knockout of *Atg7* and *Yap* in mice resulted in a phenotype with fewer liver changes than the single knockout of *Atg7*. While deletion of *Atg7* caused the development of carcinoma due to hepatomegaly, lobular and portal inflammation, ductular reaction, steatosis, and fibrosis, all these pathological signs were significantly ameliorated by removal of *Yap*, resulting in less hepatocarcinogenesis [176]. Of note, in mice with double *Atg7/Yap* knockout, carcinogenesis was attenuated despite increased expression of SQSTM1 and Nrf2, which may have mediated the residual tumorigenesis observed in double KO livers. These results suggest that YAP is an independent driver of HCC. Based on the data obtained, the tumor suppressive effect of autophagy in the liver is dependent on the inhibition of YAP-mediated cell dedifferentiation, inflammation, fibrosis, and promotion of hepatocarcinogenesis. This suggests an important role of YAP in all phases of liver carcinogenesis [176].

In summary, the studies reported here demonstrate that YAP may provide a link between autophagy impairment and tumor promotion. Accordingly, experimentally induced impairment of autophagy leads to accumulation of YAP, which is associated with promotion of hepatocarcinogenesis.

### YAP-mediate suppression of hepatocarcinogenesis by cell competition

It is well known that activation of YAP and TAZ promotes cancer cell growth in humans and mice [177]. Interestingly, Moya et al. [178] recently showed that the two Hippo coactivators can also exert a tumor suppressive function (Fig. 6 C). Using different mouse models, they found that liver tumor development was associated with upregulation of Yap and Taz in tumor cells and peritumoral hepatocytes. On the other hand, both Hippo coactivators were barely detectable in hepatocytes from normal liver. Analysis of human liver biopsies confirmed the accumulation of YAP and TAZ in peritumoral hepatocytes from ∼50% HCCs and intrahepatic cholangiocarcinomas (CCAs) but not in hepatocytes from healthy human livers. In addition, YAP accumulated in peritumoral zones of human colorectal carcinomas and melanoma metastases in the liver. Interestingly, targeted deletion of *Yap* and *Taz* in mouse peritumoral hepatocytes accelerated tumor cell proliferation. Conversely, experimental hyperactivation of *Yap* in peritumoral hepatocytes triggered regression of primary liver tumors and melanoma-derived liver metastases. Tumor elimination was the result of YAP/TAZ-induced nonapoptotic programmed cell death in tumor cells. All these data suggest a competitive interaction between tumor cells and their surrounding tissues. Whereas tumor cells from wild-type livers required YAP and TAZ for their survival, tumor cells surrounded by *Yap-* and *Taz-*deficient hepatocytes did not rely on the expression of either coactivator. This suggests that the Hippo coactivators act through a mechanism of cell competition to eliminate tumor cells. The phenomenon of “cell competition,” originally described in Drosophila [179], relies on the elimination of intact viable cells from a tissue when they are adjacent to cells with higher fitness (so-called loser and winner cells) [180]. The winner or loser status can vary depending on changes in adjacent cells. For example, Moya et al. found that activation of YAP/TAZ is not an absolute requirement for liver tumor cell survival, but rather depends on the levels of YAP/TAZ in neighboring hepatocytes. Tumor cells die when peritumoral hepatocytes have higher YAP/TAZ activity but survive when competition is neutralized, for example, when both tumor cells and peritumoral hepatocytes delete or overexpress *Yap/ Taz*.

From these data, it appears that a key function of YAP/TAZ in tumor cells is to increase their competitive ability to protect them from the tumor suppressive effect of peritumoral cells.

## Therapeutic perspectives

The Hippo signaling pathway and its downstream effectors YAP and TAZ play a crucial role in controlling hepatocellular proliferation after injury, by tightly regulating the balance between its activation and termination by apoptosis [181]. Therefore, inactivation of the Hippo pathway and activation of YAP/TAZ could be considered as crucial targets to improve liver regeneration in organs with low or compromised regenerative capacity, such as the liver of chronically diseased patients. In these cases, it may be extremely important from a clinical perspective to counteract the effects associated with chronic disease and promote the regenerative response. Nevertheless, the idea of activating YAP/TAZ to promote tissue regeneration raises safety concerns, as sustained activation of YAP/TAZ in adult mice can lead to abnormal cell proliferation, tissue fibrosis, and tumorigenesis [128, 182, 183]. Therefore, as recently extensively detailed by Moya et al. [184], several therapeutic strategies have been proposed to modulate Hippo signaling in different organs devoid of harmful side effects. Experimental approaches to efficiently modulate Hippo pathway in the liver consist of reversibility of YAP/TAZ-driven phenotypes and transient YAP/TAZ activation, hypomorphic deregulation of YAP signaling, and activation of selected YAP/TAZ target genes. Transient activation of YAP/ TAZ may allow liver regeneration without causing liver overgrowth and tumorigenesis. For example, it has been reported that although doxycycline (DOX)-inducible YAP-1SA overexpression results in massive hepatomegaly in adult mice, blocking of YAP overexpression by DOX withdrawing induces cell death in the enlarged livers, which returned to near normal size after only 2 weeks [55, 56]. Furthermore, siRNA-induced YAP deregulation in MST1/MST2 mutant livers led to regression of hepatic tumors associated with long-term YAP hyperactivation and reactivation of a hepatocyte differentiation signature [185]. In addition to temporally restricted activation, hypomorphic (partial) activation of YAP/TAZ can elicit tissue regeneration response devoid of adverse side effects. Genetic disruption of the Hyppo pathway components is known to lead to activation of YAP and, eventually, tumor formation [50, 182]. Nevertheless, the partial inhibition of Hippo kinases by pharmacological approaches allows partial activation of YAP/TAZ which could be therapeutically effective without causing negative side effects, such as tissue overgrowth and tumorigenesis. For example, administration of the MST1/2 inhibitor XMU-MP-1 (4-((5,10-dimethyl-6-oxo-6,10-dihydro-5H-pyrimido[5,4-b]thieno[3,2-e][1,4]diazepin-2-yl)amino)benzenesulfonamide) has been reported to increase liver repair and regeneration in both acute and chronic liver injury mouse models, causing a much weaker liver overgrowth phenotype than observed following genetic deletion of Mst1/Mst2 [186]. Similarly, silencing of MST1/MST2 genes could only partially activate YAP but stimulate liver regeneration [187]. Another way to avoid YAP/TAZ-associated adverse side effects is to activate their target genes. Activation of selected YAP/TAZ target genes, e.g., *Cyr61*, might be sufficient to mimic the stimulation of liver regeneration by these transcription factors. *Cyr61* (*Ccn1*) encodes a secreted cysteine-rich protein of the CCN family that regulates diverse biological processes such as cell migration, cell proliferation, and cell adhesion. In mouse liver, overexpression of the *Cyr61* gene or administration of the purified protein were shown to improve resolution of injury-induced fibrosis [188]. These results suggest that identification of YAP/TAZ target genes involved in liver regeneration may be useful for developing therapeutic strategies free of YAP/TAZ-associated tumorigenic effects.

As discussed by Moya et al. [184], although the findings here reported are exciting there is a need to exploit the potential of YAP/TAZ for regenerative medicine: (i) to clarify whether primary human cells and organs can respond to YAP/TAZ activation as observed in animal models; (ii) to evaluate the potentially deleterious effects of transient activation of YAP/TAZ in human tissues and its implication on human health; and (iii) to better clarify the role of YAP/TAZ in tissue regeneration to potentially identify genes and processes under their control that may be useful for developing new therapeutic strategies devoid of the deleterious effects related of YAP/TAZ overactivity.

## Conclusions

The Hippo signaling pathway plays a critical role in regulating liver size by modulating hepatocyte and biliary cell development [48, 66, 67]. In vivo and in vitro studies in mouse models have shown that during development and in the adult liver, activation of YAP/TAZ is required to promote proliferation of biliary epithelial cells and differentiation of hepatoblasts into biliary epithelial cells, while blocking the conversion of hepatoblasts into hepatocytes [53, 54] and maintaining hepatocytes in a quiescent state. Although YAP and TAZ are not essential to achieve proper liver size in unstressed adult livers, their activation is required for an adequate regenerative response after liver injury [64, 65, 117, 119, 171]. Hepatocytes from 2/3 hepatectomized mice with hepatocyte-specific deletions of Yap or Taz have defective cell cycle entry and progression and a decreased proliferation [51, 52]. These findings suggest that YAP and TAZ play a key role in promoting hepatocyte proliferation during liver regeneration. Better characterization of the molecular events associated with the impaired liver regeneration response in *Yap/Taz* double KO mice after tissue injury has shown that *Yap/Taz* does not exert a cell-autonomous and instructive role in hepatocytes [69]. Rather, the two Hippo coactivators are required in BECs to maintain bile duct integrity and prevent cholestasis which impairs liver regeneration by affecting immune cell recruitment and function and hepatocyte proliferation [69] (Fig. 4 A). Since YAP/TAZ are transiently activated in hepatocytes in response to liver injury, their minimal requirement in hepatocytes during liver regeneration after toxic injury is currently under debate. As discussed by Verboven et al. [69], although YAP/ TAZ may act redundantly with other signaling pathways involved in liver regeneration [2], they may also increase the competitive ability of uninjured hepatocytes, thereby selecting the fitter cells for proliferation [178] or may prime hepatocytes for trans-differentiation into liver progenitor cells or BECs [70]. The studies reported here have shown that 2/3 PH induces structural and functional changes in liver tissue architecture, including expansion of the apical surface of hepatocytes which form the bile canalicular network, and increase in acto-myosin contractility. These changes are promoted by bile acid overload induced by tissue resection and are sensed by YAP, which is localized in the F-actin-rich region of the hepatocyte apical membrane [77]. Consequently, the activated YAP translocates to the nucleus and promotes liver regeneration by mediating the expansion of the bile canalicular network through the hepatocyte dedifferentiation into HPCs which can be trans-differentiated into biliary epithelial cells (BECs) (Fig. 4 B).

Although not important for CCl4-induced liver regeneration [189], such YAP/TAZ-mediated trans-differentiation of hepatocytes into BECs could promote hepatocyte plasticity, which enable trans-differentiation of hepatocytes in various types of liver injury [69]. Many factors have been shown to promote recovery of liver mass after injury through the activation of YAP/TAZ [80, 85, 97], suggesting their cooperation with several signaling pathways. The network analysis approach used on liver regeneration has revealed complex interactions between the Hippo pathway and other signaling pathways, including TNF-α, IL-6, IFN-γ, Hedgehog, Notch, TGF-β, and WNT. These mediate the recruitment and activation of cells of the innate immune system that promote liver regeneration through the release of mitogenic cytokine [79, 134, 190–192] and phagocytosis of cell debris [69] and of HSCs which are important for the ECM reconstitution [134].

After moderate liver injury and necrosis, proportional hepatocyte proliferation occurs until the original liver mass is restored. However, in more severe injury, as occurs in chronic liver disease, the predetermined threshold for proper liver regeneration is exceeded, resulting in a repair response. Under these conditions, YAP/TAZ has been shown to promote injury healing by inducing the rapid recruitment and proliferation of a variety of resident and non-resident liver cells. The studies reported here have shown that activation of YAP/TAZ during NASH, fibrosis, and IRI is a key factor in mediating the inflammatory and wound healing responses (Fig. 5). However, there appear to be conflicting results about the role that the Hippo signaling pathway plays in modulating the innate immune response in NASH livers. While in the studies by Song and Wang [109, 112], it was shown that YAP/TAZ activation in KCs promotes NASH progression to fibrosis by inducing transcription of inflammatory cytokine genes and M1/M2 polarization (Fig. 5 A), in the study by Wang et al. [113] it was found that Nrf2-dependent YAP activation in KCs attenuated disease progression and inflammation in NASH livers by inhibiting the NLRP3 inflammasome and promoting the M2 anti-inflammatory phenotype (Fig. 5 B). YAP-mediated hepatoprotection was also observed in OLT patients and in mouse liver IRI model [157]. As with NASH, the immunosuppressive role of YAP during IRI was found to depend on its interaction with β-catenin, leading to inhibition of XBP1-mediated NLRP3 inflammasome activation and induction of the M2 reparative macrophage phenotype [121] (Fig. 5 B). Of note, Nrf2 signaling was required for YAP-mediated cytoprotection in both NASH and IRI models [113, 121, 157]. Therefore, YAP has been reported to both positively (by inducing transcription of inflammatory genes) and negatively (by inhibiting NLRP3 inflammasome activity) modulate the fibrogenic response in chronic liver injury, depending on the study. In addition, a recent study by Wang et al. [193] demonstrated that the cytoplasmic YAP positively regulates the action of the NLRP3 inflammasome in vitro and in vivo, further complicating our understanding. Specifically, this study reported that cytoplasmic YAP binds to NLRP3 in peritoneal mouse macrophages under LPS stimulation and promotes its stability by blocking the accessibility of the E3 ligase β-TrCP1, which mediates proteasomal degradation of NLRP3 via ubiquitination. Accordingly, activation of Hippo signaling leading to YAP degradation was associated with a marked decrease in NLRP3 inflammasome activity in vitro. From this study, YAP promotes NLRP3 expression at the posttranscriptional level. Consistent with this, the lack of YAP in LPS-stimulated mouse macrophages significantly decreased the protein expression of NLRP3 without affecting the expression of caspase-1, pro- IL -1β, and ASC. Thus, data on the effect of YAP on NLRP3 activity are also contradictory, suggesting both negative [113, 121] and positive modulation [193]. While negative modulation of NLRP3 by YAP involves its nuclear translocation leading to induction of transcriptional activation of β-catenin, positive modulation requires cytoplasmic localization of YAP and physical interaction of its C-terminal transactivation domain (residues 151-488), rather than the N-terminal TEAD binding domain, with NLRP3. Subsequently, post-translational modification of the inflammasome occurs [193]. On this basis, it could be suggested that the positive or negative modulation of NLRP3 by YAP is due to the different context of liver injury, which may lead to YAP phosphorylation at different sites, which in turn determine its cell localization and consequent activities.

During fibrosis, YAP mediates Cyr61-dependent recruitment of macrophages [146] (Fig. 5 D) and promotes trans-differentiation of Q-HSCs to M-HSCs based on Hedgehog/YAP-mediated glutaminolysis stimulation [136] (Fig. 5 C). Modulation of the dual ability of YAP to trigger both cytoprotective and fibrogenic mechanisms may therefore be critical to maintain liver homeostasis in vivo by limiting local organ damage and inhibiting HSC-dependent fibrosis. Another postulated mechanism by which YAP/TAZ promotes injury repair in chronic liver disease is trans-differentiation phenomena between hepatocytes and biliary epithelial cells [73]. These plasticity mechanisms and modulation of the microenvironment are commonly observed in chronic injury and predispose to liver carcinogenesis [68].

**Fig. 7:**
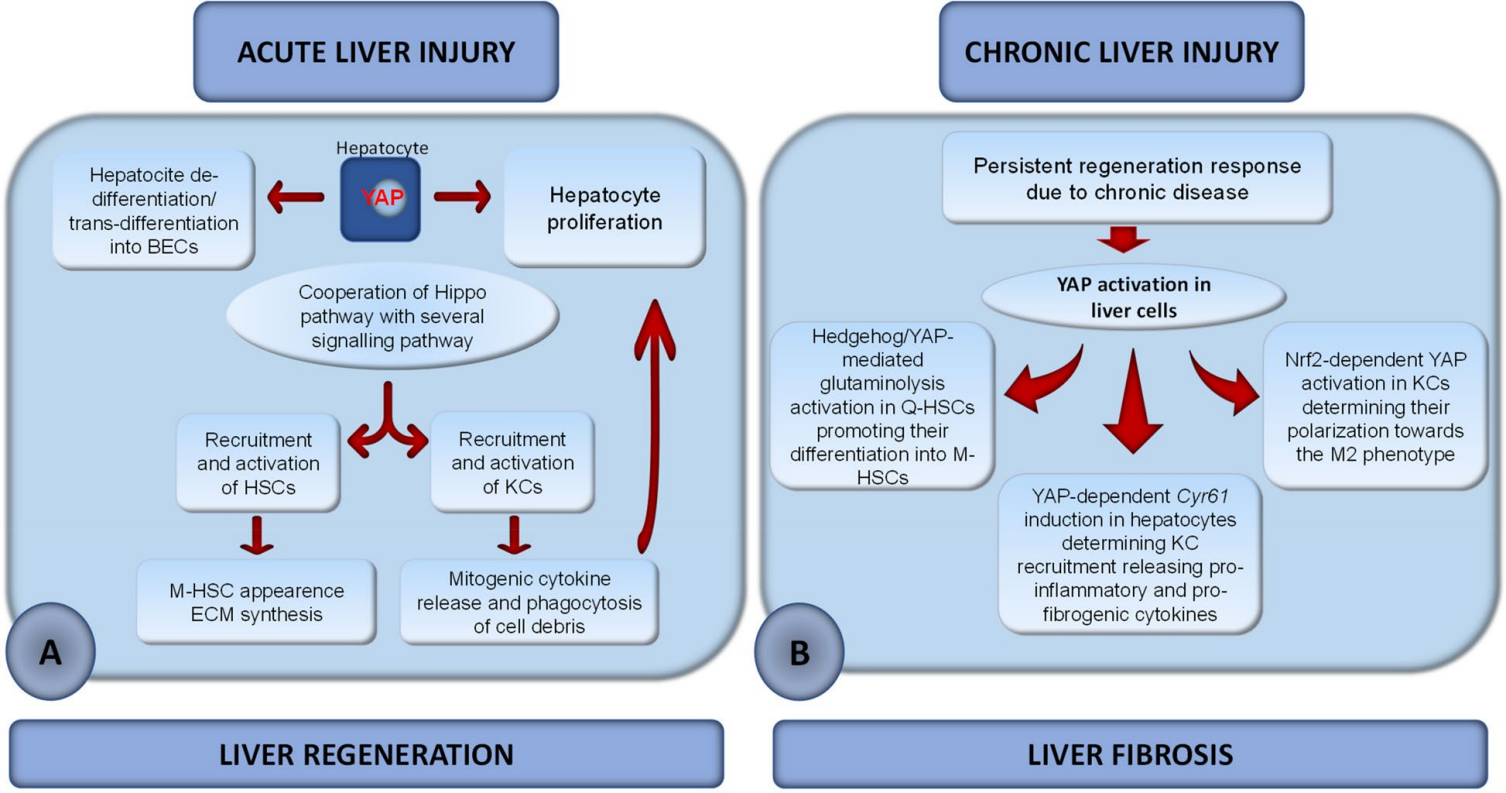
Schematic representation of the role of YAP/TAZ in normal and chronic injured liver. In normal liver (**A**), YAP activation after liver injury induces liver regeneration by promoting the formation of a new bile duct network through direct BEC proliferation or/and hepatocyte dedifferentiation to HPCs which can trans-differentiate to BECs. The creation of a new bile duct network is necessary to prevent cholestasis which impairs liver regeneration by affecting immune cell recruitment and function. Once activated in hepatocytes, YAP/TAZ signaling interacted with other signaling pathways, which mediate the recruitment and induction of KCs releasing mitogenic cytokines for parenchymal and/or non-parenchymal cells. Activated KCs also operate the phagocytosis of cell debris to allow a proper tissue regeneration. In addition, Hedeghog/YAP-mediated activation of HSCs promotes liver regeneration through ECM protein synthesis. In chronic diseased liver (**B**), where the predetermined threshold for proper liver regeneration is exceeded, YAP activation promotes a wound healing response. Indeed, chronic disease induces a persistent regeneration response in the liver, which is associated to extensive accumulation of ECM and disruption of normal hepatic structure and function. Thus, YAP activation in liver cells results in a reparative process which is characterized by liver fibrosis development, due to (i) Hedgehog/YAP-mediated glutaminolysis activation in quiescent HSCs which promote their differentiation into myofibroblastic HSCs; (ii) YAP-dependent Cyr61 gene induction in hepatocytes which determine the recruitment of macrophages in the injured liver that release inflammatory and pro-fibrogenic cytokines; (iii) Nrf2-dependent YAP activation which is associated to inhibition of NLRP3 inflammasome in macrophages determining their polarization toward the M2 phenotype associated to the release of anti-inflammatory and reparative cytokines

The role of YAP/TAZ following acute and chronic liver injuries is summarized in Fig. 7.

It has long been known that chronic liver injury is a risk factor for liver cancer [194] (Fig. 3). Since the Hippo pathway is altered in a variety of chronic liver injury, it is not surprising that up to 65% of HCCs exhibit dysregulation of the Hippo/YAP pathway, which is associated with a significantly worse prognosis [195]. Activation of YAP represents an early event in the development of liver cancer [196]; strikingly, despite extensive efforts, no germline or somatic mutations of the Hippo pathway gene have been uncovered [197]. Therefore, the mechanisms underlying YAP dysregulation during hepatocarcinogenesis are still unclear. It has been demonstrated that activation of YAP in damaged hepatocytes promotes their selective elimination by apoptosis [162] (Fig. 6 A), which may be important for maintaining genomic stability, thereby inhibiting malignant transformation. A similar protective effect against tumor development is exerted by autophagy of damaged cellular components after tissue injury [198]. The studies reported here have shown that impaired autophagy leads to liver carcinogenesis due to decreased lysosome-mediated YAP degradation [176] (Fig. 6 B). This is one reason for the therapeutic use of drugs that inhibit YAP, such as veteporfirine or carbamazepine, to attenuate hepatocarcinogenesis in chronic liver disease. Whether YAP signaling acts in synergy with other known autophagic signaling pathways, such as the Atg-dependent SQSTM1/NRF2, or as a stand-alone pathway is currently under investigation [199]. Since modulation of autophagy, especially its inhibition, is also associated with other diseases such as autoimmune and metabolic disorders, there is still debate as to which pharmacological option—induction of autophagy or inhibition of its downstream effectors (SQSTM1/NRF2, YAP)—would be the better strategy to combat liver cancer [199]. Interestingly, a recent work by Moya et al. [178] advises caution in systemic inhibition of YAP/TAZ to suppress liver carcinogenesis. Indeed, a mechanism of tumor suppression by YAP and TAZ has been described in which activation of YAP/TAZ in normal peritumoral tissue suppresses tumor growth and causes its regression (Fig. 6 C). Of note, this mechanism of tumor destruction relies on a non-cell-autonomous action of YAP and TAZ in normal peritumoral cells rather than direct regulation of target genes in cancer cells. This raises the concern that systemic inhibition of YAP/TAZ may have adverse pro-tumorigenic effects, urging caution in the use of YAP/TAZ-repressive drugs for the treatment of liver cancer.

Given the crucial role played by YAP/TAZ in controlling liver regeneration after injury, their activation may be considered crucial to improve the regenerative response in the liver of chronically diseased patients who have reduced regenerative capacity. However, since sustained activation of YAP/TAZ in the adult liver can promote tissue fibrosis and tumorigenesis, therapeutic strategies based on YAP/TAZ activation without negative side effects are desirable [128, 182, 183]. To date, several experimental approaches have been tested in animal models to modulate YAP/TAZ activation to promote liver regeneration without side effects with encouraging results [55, 56, 184–188]. Essentially, these approaches are aimed to avoid an overexpression of YAP/TAZ, so they rely on a transient activation of YAP/TAZ or direct activation of their target genes. Nevertheless, the adoption of YAP/TAZ modulation as a therapeutic strategy for regenerative medicine requires a better characterization of the molecular mechanism associated with the regulation of the Hippo pathway during liver regeneration as well as an in-depth analysis, of the efficacy and health safety, with respect to the potential application of these strategies in human liver [184].

## Data Availability

Not applicable.

## Abbreviations

2/3 PH: 2/3 partial hepatectomy
1/3 PH: 1/3 partial hepatectomy
HCC: Hepatocarcinoma
μPA: Urokinase plasminogen activator
HGF: Hepatocyte growth factor
HB-EGF: Heparin-binding EGF-like growth factor
VEGF: Vascular endothelial growth factor
EGFR: Epidermal growth factor receptors
LSECs: Liver sinusoidal endothelial cells
TGF-α: Transforming growth factor α
FGF1 and 2: Fibroblast growth factor 1 and 2
GM-CSF: Granulocyte-macrophage colony-stimulating factor
TNF-α: Tumor necrosis factor alpha
IL-6: Interleukin
STAT3: Signal Transducer and Activator of Transcription 3
JAK: Janus kinase inhibitor
C/EBPβ: CCAAT/enhancer-binding protein beta
NFκB: Nuclear Factor Kappa B
IEGs: Immediate early genes
TGF-β: Transforming growth factor beta
miRNAs: MicroRNAs
HPCs: Hepatic progenitor cells
DR: Ductular reaction
YAP 1: Yes-associated protein 1
WWTR1/TAZ: WW domain containing transcription regulator 1
MST1 and MST2: Mammalian sterile 20-like protein kinases 1 and 2
LATS1 and 2: Large tumor suppressor kinases 1 and 2
SAV1: Salvador family WW domain containing protein 1
MOB1A and MOB1B: MOB kinase activators 1A and B
TEAD: TEA domain
Ctgf 28: Connective tissue growth factor 28
Jag1: Jagged 1
Notch 2: Notch receptor 2
KO: Knock out
WT: Wild type
Ccna2/b2: Cyclin A2/Cyclin B2
ccnd1: Cyclin D1
Akt/PKB: Protein kinase B
HSCs: Hepatic stellate cells
BECs: Biliary epithelial cells
HNF4α: Hepatocyte nuclear factor-4 alpha
CCl_4_: Carbon tetrachloride
PXR: Pregnane X receptor
M1 and M2: Macrophages phenotype 1 and 2
MSCs: Mesenchymal stem cells
COL1α1: Collagen Type I Alpha 1 Chain
Cpt-1: Carnitine palmitoyltransferase 1A
Fasn: Fatty acid synthase
Areg: Amphiregulin
Snail: Snail Family Transcriptional Repressor 1
TIMP: Tissue inhibitor of metalloproteinases 1
PDGFc: Platelet-derived growth factor c
Birc5: Baculoviral IAP Repeat Containing 5
Foxm1: Forkhead Box M1
IL-1β: Interleukin 1 beta
5-HT: 5-Hydroxytryptamine
ERK: Extracellular signal-regulated kinase
Notch1: Notch receptor 1
NICD: Notch intracellular domain
SW/SNF: SWItch/Sucrose Non-Fermentable
Sox9: SRY-Box Transcription Factor 9
BET proteins: Bromodomain and extraterminal proteins
BRD4: Bromodomain-containing protein 4
JQ1: (S)-tert-butyl2-(4-(4-chlorophenyl)-2,3,9-trimethyl-6H-thieno[3,2-f][1,2,4]triazolo[4,3-a][1,4]diazepin-6-yl) acetate
Rbpj: Recombinant signal binding protein for immunoglobulin k region
IQGAP1: IQ motif containing GTPase activating protein 1
Cyr61: Cysteine-rich protein 61
Brg-1: Brahma-related gene 1
CK19: Cytokeratin19
NAFLD: Non-alcoholic fatty liver disease
NASH: Nonalcoholic steatohepatitis
RDCs: Reactive appearing ductular cells
Shh: Sonic hedgehog
KCs: Kupffer cells
HFD: High-fat diet
LPS: Lipopolysaccharide (LPS)
TLR4: Toll-like receptor 4
MCP-1: Monocyte chemoattractant protein-1
Nrf2: Erythroid-derived 2-like 2 factor
NLRP3: NLR family pyrin domain containing 3
ROS: Reactive oxygen species
BMDMs: Bone marrow-derived macrophages
PA: Palmitic acid
XBP1: X-box binding protein 1
ECM: Extracellular matrix
ω -3 PUFAs: Omega-3 polyunsaturated fatty acids
α-SMA: α-smooth muscle actin
Q-HSCs: Quiescent HSCs
M-HSCs: Myofibroblastic HSCs
α-KG: Alpha-ketoglutarate
Gls1: Glutaminase1
MDI: Inducer of fibrosis reversion
PNPLA3: Patatin-like phospholipase domain-containing protein 3 or adiponutrina
VP: Veteporfirin
sALT: Serum alalnine transaminase
OLT: Orthotopic liver transplantation
IRI: Ischemia riperfusion injury
I/R: Ischemia/reperfusion
PGE2: Prostaglandine E2
Arg1: Arginase 1
LESCs: Liver endothelial sinusoidal cells
SA β-gal: Senescence-associated marker β-galactosidase
CDC42: Cell division control protein 42 homolog
Rac: Rac Family Small GTPase
ECT2: Epithelial Cell Transforming 2
FGD3: FYVE, RhoGEF and PH domain containing 3
SQSTM/p62: Sequestosome-1
KEAP-1: Kelch-like protein 1
Atg7: Autophagy-related 7
CCAs: Cholangiocarcinomas
